# Genome-Wide Association Analysis for Triazole Resistance in *Aspergillus fumigatus*

**DOI:** 10.3390/pathogens10060701

**Published:** 2021-06-04

**Authors:** Yuying Fan, Yue Wang, Gregory A. Korfanty, Meagan Archer, Jianping Xu

**Affiliations:** Department of Biology, McMaster University, Hamilton, ON L8S 4K1, Canada; fany8@mcmaster.ca (Y.F.); wangy660@mcmaster.ca (Y.W.); korfanga@mcmaster.ca (G.A.K.); archermm@mcmaster.ca (M.A.)

**Keywords:** Aspergillus, triazoles, itraconazole, voriconazole, *Aspergillus fumigatus*, antifungal resistance, whole-genome sequencing, comparative genomics

## Abstract

*Aspergillus fumigatus* is a ubiquitous fungus and the main agent of aspergillosis, a common fungal infection in the immunocompromised population. Triazoles such as itraconazole and voriconazole are the common first-line drugs for treating aspergillosis. However, triazole resistance in *A. fumigatus* has been reported in an increasing number of countries. While most studies of triazole resistance have focused on mutations in the triazole target gene *cyp51A*, >70% of triazole-resistant strains in certain populations showed no mutations in *cyp51A*. To identify potential non-*cyp51A* mutations associated with triazole resistance in *A. fumigatus*, we analyzed the whole genome sequences and triazole susceptibilities of 195 strains from 12 countries. These strains belonged to three distinct clades. Our genome-wide association study (GWAS) identified a total of six missense mutations significantly associated with itraconazole resistance and 18 missense mutations with voriconazole resistance. In addition, to investigate itraconazole and pan-azole resistance, Fisher’s exact tests revealed 26 additional missense variants tightly linked to the top 20 SNPs obtained by GWAS, of which two were consistently associated with triazole resistance. The large number of novel mutations related to triazole resistance should help further investigations into their molecular mechanisms, their clinical importance, and the development of a comprehensive molecular diagnosis toolbox for triazole resistance in *A. fumigatus*.

## 1. Introduction

*Aspergillus fumigatus* is an opportunistic human fungal pathogen that is found in a broad range of substrates and is capable of surviving and growing in numerous environmental conditions. *A. fumigatus* is the primary cause of invasive aspergillosis, a life-threatening mold infection with high morbidity and mortality rates in immunocompromised patients. Its high sporulating capacity contributes to the environmental prevalence of *A. fumigatus*, leading to the high likelihood of infection in at-risk populations [1]. Globally, it is estimated that over 200,000 cases of invasive aspergillosis occur annually [2]. However, this number may represent only one-half of actual cases due to under- and mis-diagnoses [2]. Depending on factors such as population of patients, site of infection and antifungal management, mortality rates associated with invasive aspergillosis range from 60 to 90% [3].

Currently, there are four main classes of antifungals for aspergillosis treatment: azoles, polyenes, echinocandins, and allylamines. Among all antifungal agents, aspergillosis is commonly treated with triazole antifungals as the first choice because their use has been associated with better clinical response, less infusion-related toxicity, less nephrotoxicity and increased survival [4]. For aspergillosis treatment, itraconazole and voriconazole are among some of the commonly used triazole antifungals. Triazole antifungals work by inhibiting a vital enzymatic step in the synthesis of ergosterol, a major sterol and crucial part of the fungal cellular membrane [5]. Ergosterol plays a key role in membrane fluidity, membrane permeability, the activity of membrane proteins, and cell growth [5]. The triazoles work by inhibiting the demethylation of precursor sterols by binding to 14α-lanosterol demethylase (also known as Cyp51), a crucial enzyme involved in the ergosterol biosynthesis pathway. Triazoles act as competitive Cyp51 inhibitors through the binding of the N4 in their azole ring with the heme iron atom at the center of Cyp51 [5]. This binding prevents access of precursor sterols to the active site where demethylation occurs. Disruption of this enzymatic step causes significant damage to the cell membrane and results in the accumulation of toxic sterol intermediates, eventually leading to cell lysis and death [6]. However, the emergence of triazole-resistant *A. fumigatus* strains throughout the world has been a growing public health concern and a problem in the treatment of patients with aspergillosis.

Triazole-resistant strains have been extensively documented and characterized within multiple countries around the world. The majority of these studies have focused on the prevalence of resistant strains in a clinical setting. Furthermore, most analyses of the mechanisms of triazole resistance have focused on mutations in *cyp51A,* the gene coding for the triazole target enzyme [7]. The most common mutations in *cyp51A* among clinical-resistant strains, that develop during aspergillosis treatment, occur in amino acid sites G54, G138, M220, and G448 [7,8,9,10]. Meanwhile, the most common triazole drug resistant mutations in the global *A. fumigatus* population are TR_34_/L98H and TR_46_/Y121F/T289A, with many of these resistant strains originating from the environment [11,12].

The global population structure of *A. fumigatus* is shaped by high levels of gene flow between different populations [13,14]. Triazole-resistant *A. fumigatus* genotypes can rise and spread as a result of local selection due to elevated antifungal pressure within the environment. Clonal expansion of these highly fit triazole-resistant genotypes has been suggested to have led to their high abundance across the world [15,16]. Two main factors could have facilitated the spread of *A. fumigatus* genotypes and drug-resistant genes among geographic populations: the high dispersal ability of its asexual spores by wind and contemporary anthropogenic influences such as travel and trade [1]. Additionally, as aspergillosis is one of the leading causes of fungal deaths in avian species, bird migration may also be a factor in *A. fumigatus* dispersal [17,18]. The study by Ashu and colleagues further noted a large number of triazole-resistant genotypes and determined certain resistance genotypes were more commonly found in certain population genetic clusters than others [13]. Specifically, their analyses of 2026 *A. fumigatus* strains from 13 countries revealed that certain-resistant genotypes were mostly clustered into one genetic population and it was suggested that clonal expansion might have contributed to such a distribution. 

Many research laboratories and hospitals around the world have been tracking the distribution of triazole-resistant clinical and environmental strains [19]. When examining prior epidemiological data, an increasing trend of triazole-resistant strains and infections has been reported. For example, within the Netherlands, the number of triazole-resistant infections has increased from 7.6% in 2013 to 14.7% in 2018 [20]. Another study in the Netherlands has also reported an increasing resistance rate in Radboud University Medical Center, from 0.79% between 1996 and 2001 to 7.04% between 2012 and 2016 [21]. These upward trends have also been reported in Iran (3.3% in 2013 to 6.6% in 2015), the United Kingdom (0.43% between 1998–2011 to 2.2% between 2015–2017), and in Texas, United States (7.2% between 1999–2002 to 22.6% between 2003–2015) [22,23,24]. The increasing prevalence of triazole-resistant *A. fumigatus* strains has become a major burden to many health institutions.

Triazole resistance in *A. fumigatus* is typically separated into two main categories, Cyp51A-mediated and non-Cyp51A-mediated mechanisms of resistance. In addition, studies on triazole resistance have mainly focused on three molecular mechanisms: (i) mutations in the Cyp51A protein, (ii) overexpression of the Cyp51A protein, and (iii) upregulation of drug efflux pumps. Alterations in Cyp51A are the most commonly studied mechanism for triazole resistance. Until 2008, all reported triazole resistance focused on the context of mutations in *cyp51A*. However, from 2008 onwards, the frequency of-resistant strains with no mutations in *cyp51A* was increasing [25,26]. At present, most epidemiological studies of triazole resistance in *A. fumigatus* only investigate mutations at the *cyp51A* gene. Consequently, mutations in other genes remain largely uncharacterized.

Microbial genome-wide association studies (GWAS) are a relatively new but powerful tool in understanding the relationships between genetic variations and microbial phenotypes. There have been several successful GWAS applications in identifying novel genomic markers responsible for antifungal drug resistance, with several studies focused on examining azole resistance in plant fungal pathogens [27,28]. In *A. fumigatus*, Zhao and colleagues recently conducted a genome-wide association study for itraconazole sensitivity in non-resistant clinical isolates from Japan [29]. In the current study, a GWAS was performed for itraconazole and voriconazole resistance in *A. fumigatus* based on a global population of published genomes. The aim of the study was to determine the genetic variants associated with triazole resistance using genome-wide SNP data, with the focus placed on novel *non-cyp51A* related mutations, as well as conduct a phylogenetic analysis with 195 strains to examine the phylogenetic distributions of itraconazole and voriconazole resistance in *A. fumigatus*.

## 2. Results

### 2.1. Phylogenetic Tree

**Figure 1:**
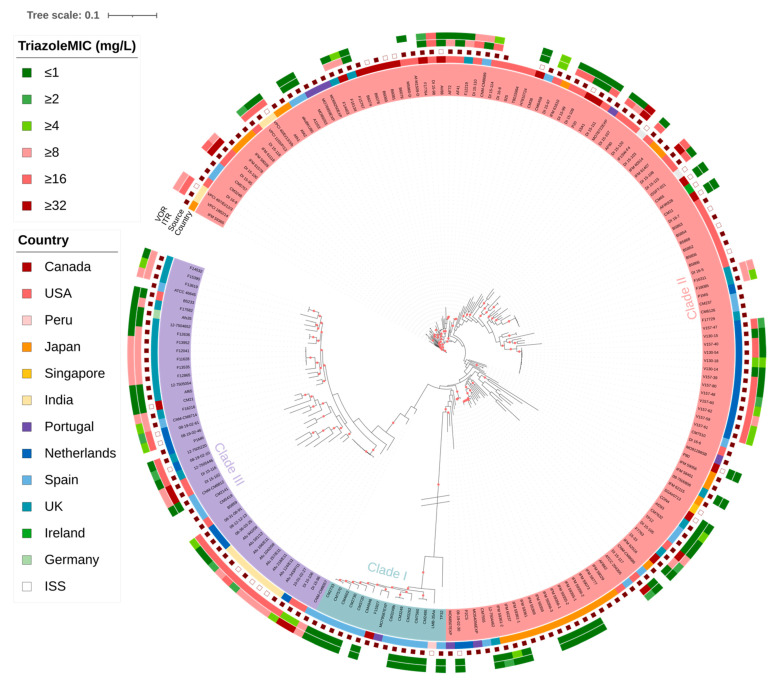
Maximum likelihood phylogenetic tree detailing the strain characteristics. Branches with a red dot represent those with over 75% bootstrap support, based on 500 bootstrap iterations. The inner-most circle denotes the clade affiliation of strains with strain names corresponding to those in Supplementary Table S1. The second inner-most circle represents country of origin for individual strains with different colors representing different countries as shown in the left “Country” panel. The third circle from the inside denotes strain ecological niche, with hollow squares representing strains from the natural environment, solid red squares representing strains from the clinical environment, and the source for the remaining strains (unmarked) were unknown. The itraconazole and voriconazole minimum inhibitory concentrations (MIC) were represented in the two outer circles with different colors representing different MIC values as shown in the left “TriazoleMIC” panel. The white boxes in the two outer circles represent strains with no MIC data. The branch length separating Clade I from the two other clades was manually truncated to make relationships in the other two clades more visible.

Phylogenetic analysis of the whole-genome SNPs grouped the 195 samples and the reference strain Af293 into three large clades based on pairwise SNP differences between all 196 strains (Figure 1). Within each clade, whole-genome SNP differences were identified as ≤35,112 in Clade I, ≤45,160 in Clade II, and ≤48,670 in Clade III. Among the analyzed strains, 15 were in Clade I, 134 strains and the reference Af293 were in Clade II, and 46 strains were in Clade III. Geographically, the Clade II strains were from 10 countries, with 16 strains found in Canada, 4 in India, 1 in Ireland, 27 in Japan, 16 in Netherlands, 7 in Portugal, 1 in Singapore, 14 in Spain, 9 in the United Kingdom, and 37 in the United States. Furthermore, two strains were collected from the International Space Station. Clade III strains were obtained from the following seven countries: Canada (n = 1), India (n = 8), Netherlands (n = 8), Spain (n = 6), the United Kingdom (n = 16), Germany (n = 1), and the United States (n = 6). Finally, the 15 Clade I strains were from four countries: Canada (n = 1), Peru (n = 1), Portugal (n = 1), and Spain (n = 12). Within each clade, the samples were predominantly from infected patients, with 93.33% (14/15) in Clade I, 82.84% (111/134) in Clade II, and 82.61% (38/46) in Clade III of the analyzed samples were from clinical sources. The overall percentage of isolates from patients in the whole sample-set was 83.59%. According to the European Committee on Antimicrobial Susceptibility Testing (EUCAST), the MIC breakpoints for susceptible strains was set at ≤1 mg/L and the area of technical uncertainty (ATU) was set at 2 mg/L for both itraconazole and voriconazole antifungals. Among the samples with available MIC information and using an MIC ≥ 2 mg/L as the resistance cut-off value for both antifungals, 61.48% (75/122) were itraconazole resistant and 43.90% (54/123) were voriconazole resistant. At a clade-level, the percentages of itraconazole-resistant strains were as follows: 0% in Clade I, 57.75% in Clade II, and 82.93% in Clade III. For voriconazole, the percentage of resistant strains were 0% in Clade I, 37.50% in Clade II, and 65.85% in Clade III. Using MICs ≥ 4 mg/L as the resistance cut-off value for both antifungals, the itraconazole-resistant rate remained the same in our sample set, at 61.48% (75/122). However, the voriconazole-resistant rate dropped to 34.96% (43/123). The percentages of itraconazole-resistant strains in each clade remained the same while the voriconazole-resistant rates were as follows: 0% in Clade I, 27.78% in Clade II, and 56.10% in Clade III. Information on the 195 strains and clade divisions can be found in Supplementary Table S1.

### 2.2. Known Mutations Associated with Triazole Resistance

The MIC data for triazole drugs in this population identified 122 and 123 strains with known itraconazole and voriconazole MIC values respectively. We first examined the statistical association between mutations at 44 amino acid sites that had been previously found to be related to triazole resistance in *A. fumigatus*. The 44 known sites were mainly identified based on epidemiological surveys and are listed in Table 1.

Among these 44 known amino acid sites, 22 SNPs at 20 amino acid positions were found in our sample-set using the filtered vcf file, prior to multiallelic site removal (Table 1). Fisher’s Exact tests were conducted on these sites to determine their statistical associations with triazole resistance (Table 1). For these tests, using the 122 strains with known MIC values for both antifungals, we identified SNPs significantly associated with itraconazole and pan-azole resistance (Table 1).

According to EUCAST guidelines, MIC breakpoints for susceptible strains are set at ≤1 mg/L with an ATU of 2 mg/L for both itraconazole and voriconazole. To accommodate this buffer region, two resistance criteria were used and tested in this study. The first test defined resistant strains as having MIC values ≥ 2 mg/L and the second test set the resistance values at MIC ≥ 4 mg/L. A Bonferroni-corrected *p*-value threshold of 4.07 × 10^−4^ (0.05/122) was used to evaluate associations between the 22 SNPs and triazole resistance. Of the 22 known SNPs tested, only one in the Lysine-98 amino acid site, located in the gene *cyp51A*, was found to be significantly associated with itraconazole and pan-azole resistance in both Fisher’s Exact tests (Table 1).

We further sought to conduct Fisher’s Exact tests using subgroups consisting of solely itraconazole resistant (i.e., resistant to itraconazole but susceptible to voriconazole) or solely voriconazole resistant (i.e., resistant to voriconazole but susceptible to itraconazole) strains groups. However, the sample sizes of these subgroups were all below the requirement needed to achieve the desired Bonferroni-corrected *p*-value threshold. Thus, these subgroups were omitted from testing.

To unmask the effect of all these listed known mutation sites in *cyp51A* associated with triazole resistance, our study conducted a stepwise analysis of these sites using Fisher’s Exact tests. First, additional Fisher’s Exact tests were conducted after strains with the well-documented L98H mutation in *cyp51A*, which alone with its accompanying tandem repeat TR_34_ can confer triazole resistance, were removed. From the 122 strains with known MIC values, 21 strains contained the TR_34_/L98H mutation (Table S1). Using both MIC resistance thresholds and a Bonferroni-corrected threshold of 4.95 × 10^−4^ (0.05/101), the additional Fisher’s Exact tests identified no SNPs significantly associated with itraconazole and/or pan-azole resistance among these 22 known mutations.

To unmask the effect of other known *cyp51A* mutations associated with triazole resistance, additional Fisher’s Exact tests were also conducted after removal of strains containing any of these known mutations (Table S1). From the strains with known MIC values, 64 strains contained the known mutations in these *cyp51A* sites (Table S1). After removal of the 64 strains and using a Bonferroni-corrected threshold of 8.62 × 10^−4^ (0.05/58), the additional Fisher’s Exact tests identified no SNPs significantly associated with itraconazole and/or pan-azole resistance among these 22 mutation sites.

A final set of Fisher’s Exact tests were conducted focusing on a clade-level. Clade II was chosen for these additional analyses as the cluster contained the greatest number of strains and none of the Clade II strains contained the L98H mutation in *cyp51A*. The strains from Clade II with both itraconazole and voriconazole MIC values (n = 71) were used in this final set of the Fisher’s Exact tests. Using a Bonferroni-corrected threshold of 7.04 × 10^−4^ (0.05/71), no SNPs were found to be significantly associated with itraconazole and/or pan-azole resistance from these 22 mutations sites.

Together, the stepwise analyses results revealed that these well-characterized mutation sites do not account for the observed triazole resistance in our sample sets. Therefore, additional modes of action and uncharacterized novel mutations should be investigated for their possible involvement with triazole susceptibility in *A. fumigatus.*

### 2.3. Genes Overexpressed with Triazole Exposure

We further examined the potential overlap between the genome-wide population level SNPs identified here with previously identified genes not listed in Table 1 but were related with triazole resistance in *A. fumigatus*. Specifically, we extracted information about specific genes that were overexpressed in *A. fumigatus* during exposure to itraconazole and/or voriconazole. Table 2 summarizes the genes that were overexpressed upon exposure to each antifungal. The overexpression of these genes under triazole stress were determined using RT-qPCR and RNA-seq information [25,38,39]. Supplementary Table S2 describes the details on the experimental conditions and setup associated with each gene listed in Table 2. Specifically, previous work demonstrated that ten ATP-binding cassette (ABC) transporters (*abcA-1*, *abcA-2*, *abcB*, *abcC*, *abcD*, *abcE, atrF*, *mdr1*, *mdr4*, and *AFUA_5G02260*), four major facilitator superfamily (MFS) transporters (*AFUA_2G11580*, *mfs56*, *mfsA* and *mfsC*), the 14-alpha sterol demethylase *cyp51A,* and 16 transcription factors (*ace1, AFUA_1G02870, AFUA_1G04140, AFUA_1G16460, AFUA_2G01190, AFUA_3G09130, AFUA_4G06170, AFUA_4G13600, AFUA_5G02655, AFUA_5G06350, AFUA_5G07510, AFUA_6G01960, AFUA_6G03430, AFUA_7G03910, AFUA_8G07360*, and *fumR*) were overexpressed following itraconazole exposure [21,35]. Similarly, five ABC transporters (*mdr1*, *abcB*, *abcC*, *abcD* and *abcE*), three MFS multidrug transporters (*mfsA*, *mfsB* and *mfsC*), a F-box domain protein (*fbpA*), an AAA-family ATPase (*aaaA*), a C6 zinc finger domain protein (*finA*), a BZIP transcription factor (*cpcA*), and a putative C2H2 zinc-finger transcription factor (*zfpA)* were overexpressed with voriconazole exposure [38].

A summary of the overexpressed genes and specific fold changes, revealed in previous studies by RT-qPCR and RNA-seq during triazole exposure, are detailed in Table 2. Using these studies, a total of 37 overexpressed genes with triazole exposure were identified for further investigation. However, subsequent analysis excluded *cyp51A*-related mutations as they have already been extensively searched and discussed in Section 2.2.

We identified SNPs in these 37 overexpressed genes and their neighbouring intergenic regions using the soft-filtered vcf file. A total of 3230 SNP sites were identified in these overexpressed genes from our dataset. Using the same procedure as that used for the 22 known mutation sites, we identified SNPs significantly associated with itraconazole-resistance and pan-azole resistance in these 36 overexpressed genes. Multiple Fisher’s Exact tests, with a Bonferroni-corrected threshold of 1.55 × 10^−5^ (0.05/3230), were conducted on these sites.

Using a MIC threshold of 2 mg/L and all 122 strains, we found 57 SNPs and 11 SNPs to be significantly associated with itraconazole and pan-azole resistance, respectively (Table S3). For itraconazole, these SNPs were located in or beside 14 genes: *abcC* (n = 9), *abcD* (n = 1), *abcE* (n = 8), *fbpA* (n = 1), *fumR* (n = 1), *mfsA* (n = 5), *mfsB* (n = 1), *mfsC* (n = 1), *AFUA_1G16460* (n = 6), *AFUA_2G01190* (n = 1), *AFUA_4G13600* (n = 3), *AFUA_5G02655* (n = 5), *AFUA_6G01960* (n = 9), and *AFUA_6G03430* (n = 6). Among these 57 SNPs, 46 were found in intergenic or intronic regions, two were non-coding transcript variants, eight were synonymous variants, and one was a missense variant (Table S3). For pan-azole resistance, the 11 associated SNPs were located in or beside six genes: *mfsA* (n = 1), *mfsB* (n = 1), *AFUA_2G01190* (n = 1), *AFUA*_*4G06170* (n = 1), *AFUA_4G13600* (n = 3), and *AFUA*_*6G03430* (n = 4). The 11 SNPs comprised of 10 intergenic variants and one missense variant. Next, using the MIC threshold of 4 mg/L as the resistance cut-off, we found 57 SNPs and 10 SNPs to be significantly associated with itraconazole and pan-azole resistance, respectively (Table S3). When compared to the previous results obtained using a MIC threshold of 2 mg/L, two variants were no longer significantly associated with pan-azole resistance: a missense variant in *mfsA* and an intergenic variant in *mfsB*. Furthermore, a newly found synonymous variant in *AFUA*_*1G04140* was significantly associated with pan-azole resistance using the MIC threshold of 4 mg/L (Table S3).

Fisher’s Exact tests were also conducted after removal of the 21 strains containing the L98H mutation in *cyp51A*. Using both MIC resistance thresholds of 2 mg/L and 4 mg/L, three SNPs were found to be significantly associated with itraconazole resistance (Table S3). The three SNPs consisted of the previously found intergenic variant in *mfsB,* and two novel intergenic variants—one in *AFUA_6G01960* and the second in *AFUA_6G01960* (Table S3). No SNPs were found to be significantly associated with pan-azole resistance.

A third set of Fisher’s Exact tests were conducted after removal of the 64 strains containing the mutations in *cyp51A* and using both MIC thresholds. Using the MIC resistance thresholds of 4 mg/L, one SNP was found to be significantly associated with pan-azole resistance. This SNP was found in the intergenic region of *abcA* (Table S3).

A final set of Fisher’s Exact tests was completed and focused solely on strains from Clade II (n = 71). Using both MIC resistance thresholds, 2 mg/L and 4 mg/L, no SNPs were found to be significantly associated with itraconazole and/or pan-azole resistance in this sample set.

### 2.4. Genome-Wide Association Study

**Figure 2:**
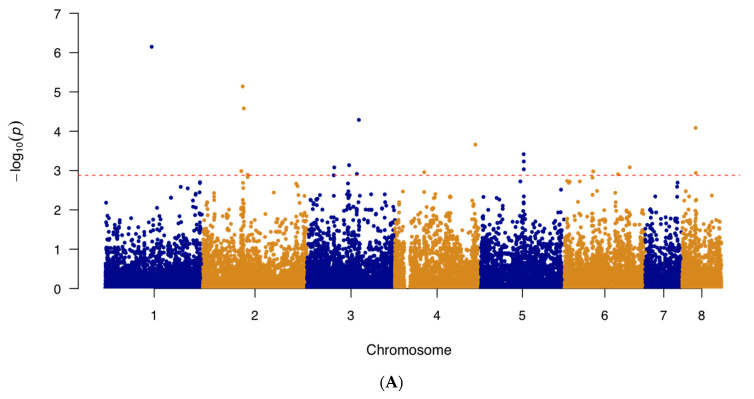

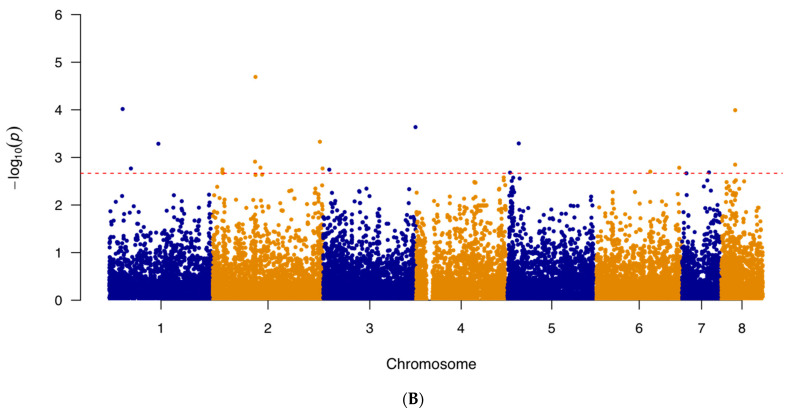
The Manhattan plot showing genome-wide SNPs associated with triazole resistance in *A. fumigatus*. (**A**) SNPs associated with itraconazole resistance in *A. fumigatus* isolates (n = 122) and (**B**) SNPs associated with voriconazole resistance in *A. fumigatus* isolates (n = 123). The top 20 SNPs in each analysis are separated out by the red dashed line. The plot is depicted with chromosome position on the X-axis and the −log_10_(*p*-value) on the Y-axis.

In addition to examining known triazole resistance mutations and SNPs in genes overexpressed during triazole exposure, a genome-wide association study (GWAS) was performed on the 122 and 123 strains with known itraconazole and voriconazole MIC values to investigate potential novel mutations associated with triazole sensitivity. The results of our analyses are summarized in Figure 2. Specifically, the itraconazole GWAS Manhattan plot can be found in Figure 2A and for voriconazole, in Figure 2B. The generated quantile–quantile plots for the GWAS results displayed no systematic inflation in our samples (Figure S1A,B).

We further examined the top 20 significant SNPs identified by the GWAS analysis. Among the 20 SNPs obtained from the itraconazole GWAS, 13 (65%) were located in intergenic regions and 7 (35%) within protein-coding regions (Table 3). These seven SNPs consisted of five missense variants, one synonymous variant, and one non-coding transcript variant (Table 3). In terms of the top 20 SNPs found from the voriconazole GWAS, 10 (50%) were found in intergenic regions and the remaining 10 in coding regions (Table 4). These 10 coding-region SNPs consist of four missense variants, five synonymous variants, and one non-coding transcript variant (Table 4). Among the top 20 SNPs associated with each of the two drugs, only one was shared. This variant was a missense A to C mutation at the position 2,538,614 on chromosome 1, in the gene *AFUA_1G09780*. The remaining 38 SNPs were unique to each of the two triazole drugs.

**Figure 3:**
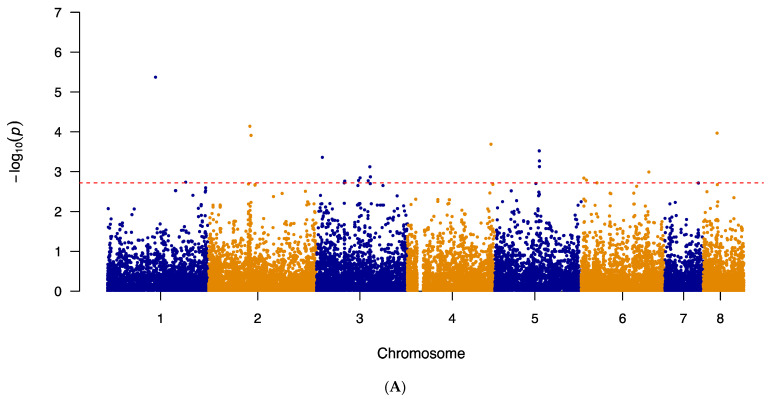

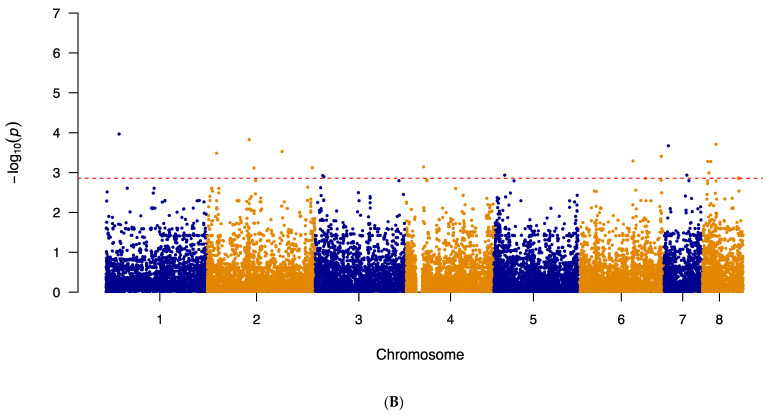
The Manhattan plot showing genome-wide SNPs associated with triazole resistance in *A. fumigatus* after removal of strains containing the L98H mutation in *cyp51A*. (**A**) SNPs associated with itraconazole resistance in *A. fumigatus* isolates (n = 101) and (**B**) SNPs associated with voriconazole resistance in *A. fumigatus* isolates (n = 102). The top 20 SNPs in each analysis are separated out by the red dashed line. The plot is depicted with chromosome position on the X-axis and the −log_10_(*p*-value) on the Y-axis.

Additional GWAS analyses were conducted in the same stepwise manner seen in the previous Fisher’s Exact tests. Firstly, to alleviate any potential masking effect caused by the L98H mutation in *cyp51A*, the 21 strains with the L98H mutation were removed. A second GWAS, using the same previous pipeline, was then conducted. The results of the second GWAS are summarized in Figure 3A,B as Manhattan plots for itraconazole and voriconazole, respectively. The generated quantile–quantile plots for both GWAS results displayed no genomic inflation (Figure S2A,B).

The top 20 significant SNPs identified by the second GWAS analyses were examined. Among the 20 SNPs obtained from the itraconazole GWAS, 13 (65%) were located in intergenic regions and 7 (35%) within protein-coding regions (Table 5). These seven SNPs comprised of four missense variants, one synonymous variant, and two non-coding transcript variants (Table 5). In terms of the top 20 SNPs obtained from the second voriconazole GWAS, 10 (50%) were found in intergenic regions and the remaining 10 in coding regions (Table 6). These 10 coding-region SNPs consist of 5 missense variants, 3 synonymous variants, and 2 non-coding transcript variants (Table 6). Among the top 20 SNPs associated with each of the two drugs, none were shared between the two triazole drugs.

**Figure 4:**
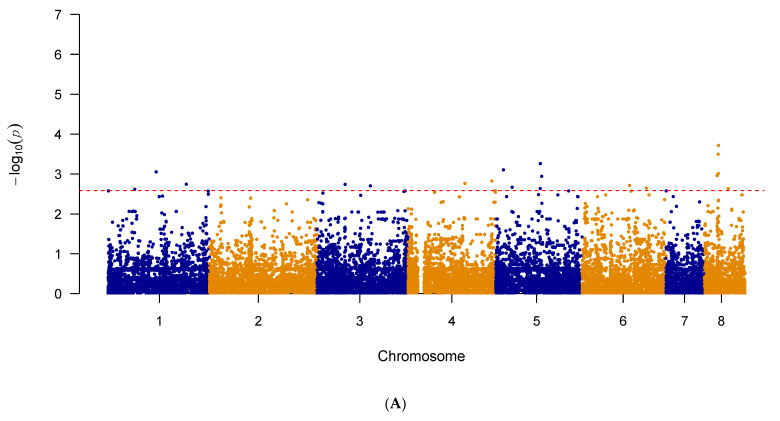

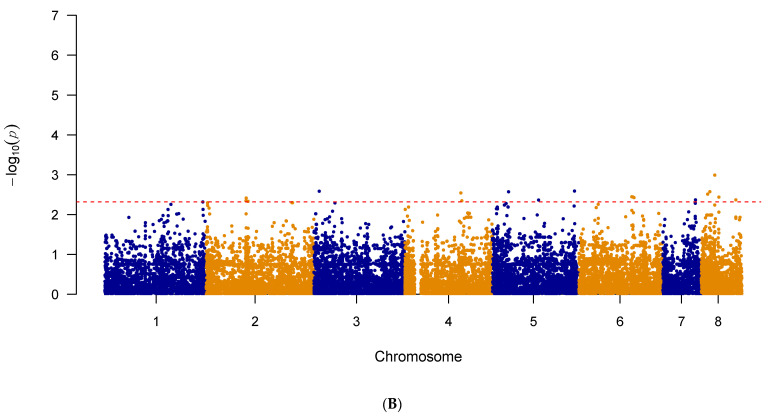
The Manhattan plot showing genome-wide SNPs associated with triazole resistance in *A. fumigatus* after removal of strains containing the mutations in *cyp51A*. (**A**) SNPs associated with itraconazole resistance in *A. fumigatus* isolates (n = 58) and (**B**) SNPs associated with voriconazole resistance in *A. fumigatus* isolates (n = 59). The top 20 SNPs in each analysis are separated out by the red dashed line. The plot is depicted with chromosome position on the X-axis and the −log_10_(*p*-value) on the Y-axis.

A third set of GWAS analyses was also done to alleviate any potential masking effect caused by the known mutations in *cyp51A,* previously listed in Table 1. The 64 strains with *cyp51A* mutations were removed and the third GWAS, using the same previous pipeline, was then conducted. The results of the third GWAS are summarized in Figure 4A,B as Manhattan plots for itraconazole and voriconazole, respectively. The generated quantile–quantile plots for both GWAS results displayed no genomic inflation (Figure S3A,B).

The top 20 significant SNPs identified by the third GWAS analyses were examined. Among the top 20 SNPs obtained from the itraconazole GWAS, 11 (55%) were located in intergenic regions and 9 (45%) within protein-coding regions (Table 7). These nine SNPs comprised of three missense variants, two synonymous variants, three non-coding transcript variants and one intragenic variant (Table 7). In terms of the top 20 SNPs obtained from the third voriconazole GWAS, 10 (50%) were found in intergenic regions and the remaining 10 in coding regions (Table 8). These 10 coding-region SNPs consist of six missense variants and four synonymous variants (Table 8). Among the top 20 SNPs associated with each of the two drugs, two SNPS were shared between the two triazole drugs. The first variant was a synonymous C to A mutation at the position 2,539,714 on chromosome 4, in the gene *AFUA_4G09770*. The second mutation was a synonymous T to C mutation at position 2,131,740 of chromosome 5, in the gene *AFUA_5G08390.*

**Figure 5:**
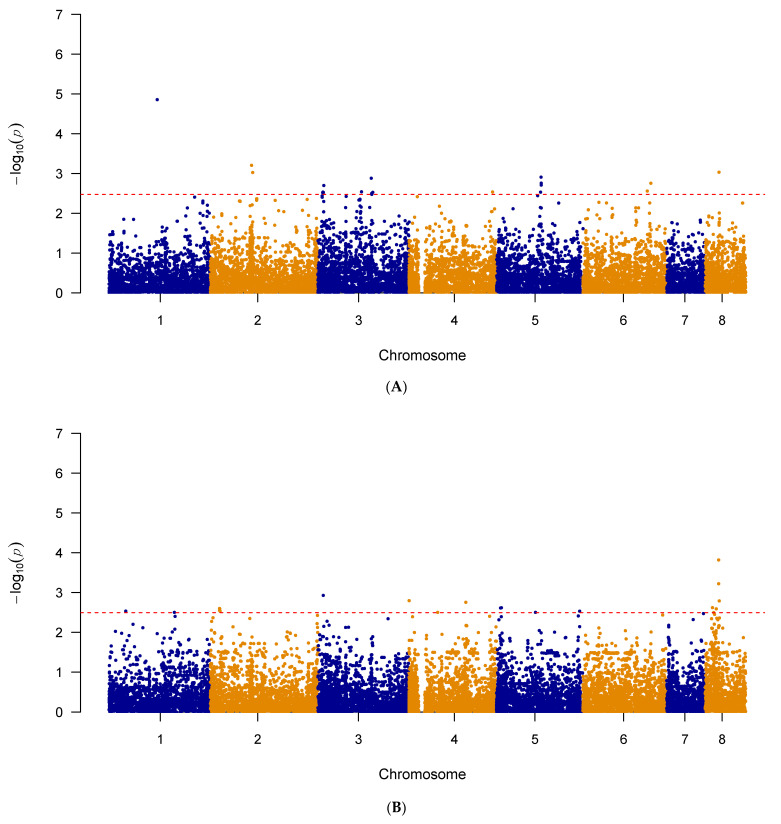
The Manhattan plot showing genome-wide SNPs associated with triazole resistance in *A. fumigatus* in Clade II (**A**) SNPs associated with itraconazole resistance in *A. fumigatus* isolates (n = 71) and (**B**) SNPs associated with voriconazole resistance in *A. fumigatus* isolates (n = 72). The top 20 SNPs in each analysis are separated out by the red dashed line. The plot is depicted with chromosome position on the X-axis and the −log_10_(*p*-value) on the Y-axis.

A final set of GWAS was completed to focus our analysis on a clade-level, using strains from Clade II. The strains from Clade II with itraconazole (n = 71) and voriconazole (n = 72) MIC values were used for the fourth GWAS, using the same previous pipelines. The results of this GWAS are summarized in Figure 5A,B as Manhattan plots for itraconazole and voriconazole, respectively. The generated quantile–quantile plots for both GWAS results displayed no genomic inflation (Figure S4A,B).

The top 20 significant SNPs identified by the GWAS analyses on strains from Clade II were examined. Among the top 20 SNPs obtained from the itraconazole GWAS, 15 (75%) were located in intergenic regions and 5 (25%) within protein-coding regions (Table 9). These five SNPs comprised of two missense variants, two synonymous variants, and one non-coding transcript variant (Table 9). In terms of the top 20 SNPs obtained from the voriconazole GWAS, 6 (30%) were found in intergenic regions and the remaining 14 in coding regions (Table 10). These 14 coding-region SNPs consist of seven missense variants, five synonymous variants, one non-coding transcript variant and one intragenic variant (Table 10). Among the top 20 SNPs associated with each of the two drugs, no mutation sites were shared between the two triazole drugs.

### 2.5. Linkage Disequilibrium Analysis

Linkage disequilibrium analyses were conducted using the top 20 SNPs obtained by the four GWAS analyses and all 314,999 SNPs in the soft-filtered vcf file to search for SNPs highly linked (R^2^ > 0.85) to these significantly associated SNPs. Specifically, we focused on highly linked non-synonymous mutations. The results of this association analysis are presented in Table 11 for itraconazole and in Table 12 for voriconazole. In total, for itraconazole resistance, we identified 15 additional highly linked missense variants located in 13 (putative) protein-coding genes (Table 11). For voriconazole resistance, this analysis revealed 11 additional missense SNPs located in 11 different (putative) protein coding genes (Table 12). None of these additional missense SNPs were shared between the two drugs.

Fisher’s Exact tests, with a Bonferroni-corrected *p*-value threshold of 4.07 × 10^−4^ (0.05/122), were conducted to examine associations among these highly linked mutations to itraconazole and pan-azole resistance (Table 13). MIC resistance thresholds of 2 mg/L and 4 mg/L were both tested for these 26 sites and using all 122 strains. Both MIC thresholds identified four SNPs to be significantly associated with itraconazole resistance as well as two of these SNPs also being associated with pan-azole resistance (Table 13).

Additional Fisher’s Exact tests were also conducted after the removal of the 21 strains with the L98H mutation in *cyp51A* and using a Bonferroni-corrected *p*-value threshold of 4.95 × 10^−4^ (0.05/101) (Table 14). For both MIC resistance thresholds, the results showed that the three previously noted SNPs, in *AFUA_1G17380, AFUA_3G09040* and *AFUA_3G09070,* were again significantly associated with itraconazole resistance. After removal of the 21 strains and using both MIC thresholds, two of these SNPs, *AFUA_3G09040* and *AFUA_3G09070,* were now also significantly associated with pan-azole resistance. Another shared SNP with the previous analysis is the missense variant in *AFUA_1G03370,* which was found to be significantly associated with pan-azole resistance at both MIC resistance thresholds. Furthermore, using the MIC threshold of 2 mg/L, a novel missense variant in *AFUA_7G06290* was found to be associated with pan-azole resistance (Table 14).

A third set of Fisher’s exact tests were conducted after removal of the 64 strains containing known *cyp51A* mutations and using a Bonferroni-corrected *p*-value threshold of 8.62 × 10^−4^ (0.05/58) (Table 15). For both MIC resistance thresholds, the tests determined three previously identified SNPs to be significantly associated with both itraconazole and pan-azole resistance. These three SNPs were a missense variant in *AFUA_1G17380, AFUA_3G09040,* and *AFUA_3G09070.* Using both MIC thresholds, the tests also identified the previous *AFUA_7G06290* missense variant to be significantly associated with pan-azole resistance.

Lastly, another set of Fisher’s Exact test was conducted to focus solely on strains from Clade II and used a Bonferroni-corrected threshold of 7.04 × 10^−4^ (0.05/71) (Table 16). For both MIC resistance thresholds, the tests determined three previously identified SNPs to be significantly associated with both itraconazole and pan-azole resistance. These SNPs were a missense variant in *AFUA_1G17380, AFUA_3G09040* and *AFUA_3G09070*. Furthermore, using both MIC thresholds, the tests also identified the previously noted missense variant in *AFUA_7G06290* to be significantly associated with pan−azole resistance (Table 16).

## 3. Discussion

In this study, we analyzed the genomic polymorphisms among 195 *A. fumigatus* isolates collected from 12 countries as well as the International Space Station to investigate the potential associations between genomic SNPs and triazole resistance. Phylogenetic analyses of the whole-genome SNPs identified three main clades in this sample, with Clade I being very divergent from the other two clades. Most strains in this clade were from Spain and they likely represent a cryptic species within *A. fumigatus sensu stricto*. Among these 195 strains, the minimum inhibitory concentrations of two triazoles, itraconazole and voriconazole, were reported for 122 and 123 strains, respectively. Over the past two decades, an increasing number of studies have been conducted to investigate the genetic diversity and population structure of *A. fumigatus* using different molecular markers [14,40,41,42,43]. A previous study exploring global population genetic variation by Ashu et al. identified 8 genetic clusters by examining nine short tandem repeats in 2026 *A. fumigatus* isolates from 13 countries [13]. However, a more recent study analyzing the same short tandem repeats of 4049 *A. fumigatus* isolates identified two broad genetic clusters [14]. The whole-genome SNP analyses here revealed three divergent clades and within both Clades II and III, several sub-clades with significant bootstrap supports were also found. Therefore, the true number and composition of the genetic clusters in the global *A. fumigatus* population remain uncertain and depend on how clades and genetic clusters are defined. However, based on previous studies, most genetic clusters and clades contain geographically and ecologically diverse strains, consistent with frequent gene flow and great adaptability of *A. fumigatus* genotypes [14,26].

Among geographic and ecological populations, different frequencies of triazole resistance have been reported, likely reflecting their variations in strain source, clinical antifungal usage, agricultural fungicide usage, and surveillance techniques [44,45,46,47,48,49,50,51]. In 2017, Garcia-Rubio et al. reviewed previously published literature and reported that the global triazole-resistant rate ranged from 0.55% to 30% [30]. In the samples analyzed here and using an MIC threshold of 2 mg/L, 61.48% of all isolates with available MIC data were itraconazole resistant and 43.90% were voriconazole resistant. Furthermore, 63.46% and 43.81% of the clinical isolates were itraconazole and voriconazole resistant, respectively. Similarly, there was a high frequency of environmental isolates resistant to itraconazole and voriconazole, at 50.00% and 44.44%, respectively. Using the MIC threshold of 4 mg/L, resistance frequencies for itraconazole remained the same, however, these values changed for voriconazole. The resistance rate for voriconazole in clinical strains decreased to 35.24% and for environmental strains, it changed to 33.33%. The high rates of resistance among strains analyzed here could be attributed to the biases among research groups in preferentially submitting drug-resistant strains for whole-genome sequencing. However, the broad range of triazole MIC values among the large number of sequenced strains allowed us to infer potential novel genetic variants not identified in previous studies.

Indeed, in this study, GWAS for both itraconazole and voriconazole resistance identified novel genes and mutations linked to triazole resistance. We conducted four GWAS analyses. The first analysis used all strains with known MIC values for itraconazole (n = 122) and voriconazole (n = 123). Meanwhile, the following two analyses investigated novel mutations associated with itraconazole and voriconazole resistance at other SNP loci, unrelated to *cyp51A*. Specifically, the second GWAS removed the 21 strains containing the L98H mutation in *cyp51A* and the third GWAS removed the 64 strains containing known mutations in *cyp51A* related to triazole resistance. The last GWAS was done on a clade-level, focusing the analysis on strains from Clade II with known triazole MIC values (n = 71). For each GWAS, we focused our investigation on the top 20 SNPs obtained via the GWAS analyses for each of the two drugs. We identified a total of six missense variants to be putatively associated with itraconazole resistance. These six missense variants were located in six genes: *AFUA_1G09780, AFUA_2G08060, AFUA_3G09090, AFUA_5G08150, AFUA_6G01860,* and *AFUA_8G02350*. The first mutation was in *AFUA*_*1G09780*, which encodes for a stomatin family protein. The stomatin proteins belong to a highly conserved family of integral membrane proteins. In humans, stomatin interacts with various ion channels and modulates their activity. The proteins are also thought to perform specific scaffolding functions in membranes. However, the functional information of stomatin proteins in fungi is scarce. The ortholog of *AFUA*_*1G09780* in the closely related *Aspergillus nidulans* species is *AN1287* (*stoB*). The AN1287 protein is located in the inner mitochondrial membrane [52]. In addition to targeting ergosterol biosynthesis, triazole exposure also causes production of deleterious mitochondrial reactive oxygen species (ROS). Therefore, since triazoles promote ROS accumulation, the mitochondrial membrane complexes represent another group of targets when studying resistance [53]. Of note, this mutation in *AFUA_1G09780*, was found to be significantly associated with itraconazole resistance in all four GWAS analyses. The second mutation was in *AFUA_5G08150*, which encodes for a putative ABC bile acid transporter. Although this specific gene has not been previously linked to triazole resistance, other ABC transporter members are known to modulate triazole extrusion. Previous studies have found multiple ABC transporter members to be overexpressed with triazole exposure and that expression levels of various ABC transporter members were higher among triazole-resistant isolates [23,37,54]. In most cases, overexpression in this family of transporters seems to prevent intracellular drug concentrations from reaching levels needed to be effective at impacting ergosterol biosynthesis. The next variant was found in *AFUA_8G02350*, encoding a putative polyketide synthase. The polyketide synthases are involved in the biosynthesis of polyketides, which are a large and structurally diverse group of secondary metabolites [55]. These compounds have a wide range of biological activities that are important for ecological and evolutionary adaptation in fungi [55]. Furthermore, the gene *AFUA_8G02350* is within a terpene hybrid cluster [56]. The fourth gene, *AFUA_3G09090*, encodes for an uncharacterized protein. The fifth missense mutation related to itraconazole resistance was found in *AFUA_2G08060*, which encodes an involucrin repeat protein. This protein has been found to be involved in tethering Woronin bodies to septal pores [57]. In multicellular fungi such as *A. fumigatus*, cells are connected to each other via intercellular bridges called septal pores and Woronin bodies plug these pores upon injury to avoid excessive loss of cell content. Deletion mutants with impaired Woronin bodies have shown to have impaired stress resistance and delayed hyphal wounding response [57]. The missense mutation identified here may enhance the strains’ ability to respond quickly to triazole drugs. The last missense mutation was in *AFUA_6G01860*, which encodes a putative MFS lactose permease. This class of protein plays a role in transmembrane transport and is an MFS family member, a superfamily of transport proteins that are mainly responsible for antifungal resistance development through drug efflux activity.

Interestingly, our itraconazole GWAS analysis results differ from those in a GWAS conducted by Zhao and colleagues who examined SNPs associated with itraconazole sensitivity [29]. They completed a GWAS using 76 clinical *A. fumigatus* isolates collected from Japan. Our comparisons revealed no overlap between our top 20 SNPs and the SNPs they found to be highly associated with itraconazole sensitivity. Two factors might have contributed to the different observations. In the first, the study by Zhao et al. focused on itraconazole sensitivity in non-resistant clinical isolates of *A. fumigatus*, with itraconazole MIC ranging from 0.125 to 1 mg/L among their 76 isolates [29]. In contrast, the itraconazole MICs for our 122 strains with itraconazole MICs ranged from 0.13 to 32 mg/L. Secondly, all the strains analyzed by Zhao et al. were from one country, Japan [29]. In contrast, our itraconazole GWAS included 122 strains from eight countries, Canada (n = 12), India (n = 12), Japan (n = 8), Netherlands (n = 21), Spain (n = 19), Germany (n = 1), the United Kingdom (n = 24), and the United States (n = 25). Together, these results suggest that additional novel SNPs associated with itraconazole sensitivity or resistance will likely be present in other geographic and/or ecological populations of *A. fumigatus*. Another recent preprint by Rhodes and colleagues also conducted a GWAS on itraconazole resistance using treeWAS, a phylogenetic tree-based GWAS approach [58]. A comparison between the top 20 SNPs from our GWAS analyses and their significantly associated SNPs to itraconazole resistance found no overlap in SNP sites. This difference in results is most likely related to sample selection, as their sample set focused on strains obtained from the United Kingdom and Republic of Ireland. Another potential reason for the discrepancy is that our study used a quantitative phenotype, based on MIC values, for our GWAS while Rhodes and colleagues used a binary phenotype, separating strains into susceptible and resistant strains by defining resistance as MIC ≥ 2 mg/L. However, their results are consistent with our general conclusion that a large number of additional novel mutations, un-related to *cyp51A*, are significantly associated with triazole resistance in *A. fumigatus*.

The voriconazole GWAS analyses identified a total of 17 missense variants to be putatively associated with resistance. These variants were found in 17 different genes: *AFUA_1G03370, AFUA_1G09780, AFUA_1G12540,* *AFUA_1G17410,* *AFUA_2G01700*, *AFUA_2G13030, AFUA_4G09580*, *AFUA_5G01000*, *AFUA_5G02210, AFUA_5G14610*, *AFUA_6G09870*, *AFUA_7G00740,* *AFUA_7G05960,* *AFUA_8G01250, AFUA_8G01340, AFUA_8G01940,* and *AFUA_8G02280*. Two of these missense variants were found in *AFUA_1G03370* and *AFUA_5G02210*, which encodes for putative proteins of unknown functions. A third gene, *AFUA_1G12540*, encodes a putative TMEM1 family protein with uncharacterized function. The next seven variants were found in members of enzyme families with roles across a large number of biological processes, which comprised of the genes *AFUA_1G17410* that encodes a putative beta-glucosidase, *AFUA_2G01700* that encodes a putative serine/threonine protein kinase, *AFUA_2G13030* that encodes a phenylalanyl-tRNA synthetase, *AFUA_5G01000* that encodes a putative oxidoreductase of the 2-oxoglutarate (2OG)-Fe(II) oxygenase superfamily, *AFUA_5G14610* that encodes a putative carboxypeptidase Y, *AFUA_7G00740* that encodes a putative protein kinase, and *AFUA_8G01250* that encodes a putative GNAT family acetyltransferase. The next variant was found in *AFUA_7G05960*, which encodes a putative C2H2 finger domain protein. Three significantly associated missense variants also encoded for putative C6 zinc cluster transcription factors, which were found in *AFUA_6G09870*, *AFUA_8G01940* and *AFUA_8G02280*. Other members of this transcription factor family have been linked to triazole resistance. For example, a previous transcriptome study had found *finA*, a C6 zinc finger domain protein, displayed increased mRNA levels during adaptation to voriconazole exposure [38]. In addition, another C6 zinc-cluster transcription factor, AtrR, had been found to be associated with triazole resistance by regulating expression of genes related to ergosterol biosynthesis [59]. However, the Zn cluster family is the largest family of transcription factors known in eukaryotes and thus additional testing is required [60]. Interestingly, a missense mutation in *AFUA_1G09780* was also found significantly associated to voriconazole and was the same SNP found to be associated with itraconazole resistance. The remaining two genes were *AFUA_8G01340* that encodes a putative MFS sugar transporter and *AFUA_4G09580,* which encodes the major allergen Aspf2. Although our study focused on examining missense variants, significantly associated SNPs obtained by GWAS also included synonymous, intergenic and intronic variants. These variants can have biological consequences and contribute to functional changes in the protein. For example, synonymous mutations can affect critical cis-regulatory sequences, alter mRNA structure, and impact translational speed [61]. Furthermore, non-coding variants can be found within potential regulatory sequences such as enhancers, promoters, and 5′ and 3′ UTRs [62]. Through these regulatory roles, non-coding variants can influence processes such as transcription, translation and splicing.

The results of the itraconazole and voriconazole GWAS showed few overlaps between significant SNPs; the first GWAS having one SNP overlap in the gene *AFUA_1G09780* and the third GWAS having two shared SNPs, a synonymous mutation in *AFUA_4G09770* and a synonymous mutation in *AFUA_5G08390.* The remaining two GWAS found no overlapping SNPs between the two antifungal drugs. Several reasons could have contributed to the low number of shared SNPs. Although all azoles operate using the same common mode of action, decreasing ergosterol synthesis by inhibiting the fungal enzyme 14α-sterol demethylase, there are differences between itraconazole and voriconazole in terms of their mechanisms of action. Voriconazole also inhibits 24-methylene dihydrolanasterol demethylation in *Aspergillus* and its antifungal activity is likely a result of a combination of effects in addition to inhibition of ergosterol synthesis [63]. Secondly, our sample set consisted of a large number of strains that had different susceptibilities to the two drugs and were only resistant to one of the two antifungals. Finally, our sample set was not a natural randomly mating population but were selected strains sequenced by different laboratories based on their own specific objective and purpose. As a result of the diversity of strains and their originating populations, some of the shared azole-resistance related mutations may have been less frequent in our studied sample set and were, thus, likely filtered out during quality control.

Linkage disequilibrium was also evaluated using the top 20 SNPs obtained by each of the four GWAS analyses to identify additional highly linked missense SNPs. Four sets of Fisher’s Exact tests were conducted: using all 122 strains, after removal of the 21 strains with the L98H mutation, after removal of the 64 strains with the *cyp51A* mutations, and using only the 71 Clade II strains. Interestingly, the last three tests identified two SNPs, missense variants in *AFUA_3G09040* and *AFUA_3G09070,* to be significantly associated with itraconazole as well as pan-azole resistance using both MIC thresholds (Table 14, Table 15 and Table 16). In the first test, conducted using all 122 strains and at both MIC thresholds, these two missense variants were also found to be significantly associated with itraconazole resistance (Table 13). Another SNP in *AFUA_1G17380* was also found in three tests to be significantly associated with itraconazole and pan-azole resistance using both MIC thresholds (Table 13, Table 15, and Table 16). Furthermore, in the remaining test, the SNP site was significantly associated with itraconazole resistance (Table 14). In terms of the function of these three genes, *AFUA_1G17380* encodes a putative 3-oxoacyl-(acyl-carrier-protein) reductase, *AFUA_3G0904* encodes for an uncharacterized protein and *AFUA_3G09070* encodes a putative carboxylesterase.

In addition, our study examined 20 previously known amino acid sites associated with triazole resistance. Fifteen of these known amino acid sites were in the *cyp51A* gene. Of these 15 sites, several have been functionally validated in previous studies and found to directly contribute to triazole resistance. These validated mutation sites consisted of G54, L98, Y121, G138, M220, T289 and G448 [8,64]. However, hot-spot SNP sites that confer triazole resistance by itself only include five of the seven sites, at G54, Y121, G138, M220 and G448. Meanwhile, for L98 and T289, a combination with a tandem repeat is required for triazole resistance, specifically TR_34_/L98H and TR_46_/Y121F/T289A [65]. Mutations in the G138 site has also been validated to cause multi-azole resistance [66]. Lastly, SNP sites P216 and F219 both confer resistance to itraconazole when mutated [67]. Specifically, there have been two amino acid substitutions in F219 in triazole-resistant strains, F219I and F219L [67]. However, interestingly, we have instead identified a different substitution F219S in our sample set; although it was not significantly associated with resistance (Table 1). We have also included the amino acid sites F46, M172, N248, D255, and E427; although only 3 of these sites could be found in our filtered genotype file, specifically F46, D255, and E427. Strains with a combination of these five mutations, as F46Y/M172V/D255E or F46Y/M172V/D255E/N248T/E427K, have shown higher triazole MICs than the wild-type strains [30]. The three remaining *cyp51A* mutation sites, H147, S297 and F495, have been identified to be associated with resistant isolates but have not been functionally validated. Mutations in H147 were found to coincide with isolates with G448 mutations and were not found to be associated with resistance by itself and is thought to only increase protein stability [9,68]. Similarly, S297 and F495 mutations are found in some TR_34_/L98H mutant *A. fumigatus* strains but have not been proven to be sufficient for triazole resistance by themselves [12,69]. However, these two amino acid positions, S297 and F495, are located near the triazole binding pocket of Cyp51A [70]. The remaining five known amino acid sites associated with triazole resistance are non-*cyp51A* mutations. Three of these SNP sites (I412, P309, and S305) are in *hmg1*, which encodes a 3-hydroxy-3-methyl-glutaryl-coenzyme A (HMG-CoA) reductase. Mutation at these three sites have been identified in triazole-resistant isolates and their association with triazole resistance has been functionally validated by inserting each SNP into the *hmg1* gene of the laboratory strain akuB^KU80^ [33]. These three sites, I412, P309, and S305, are predicted to be within the conserved sterol-sensing domain of *hmg1* [33]. Furthermore, akuB^KU80^ mutants with the substitution I412S and S305P possessed significantly different cellular sterol profiles compared to the unaltered akuB^KU80^ strain; independent of *cyp51A* and *cyp51B* expression [33]. Another known mutation site is L167 in the uncharacterized *AFUA_7G01960* gene. The nonsense mutation L167* was first identified in the multi-azole-resistant clinical isolate V157-62, and subsequently functionally validated by inserting the specific SNP into an azole-susceptible clinical isolate, V130-15 [36]. At this amino acid position, a nonsense mutation was generated, which increased resistance to itraconazole [36]. Furthermore, this SNP was also associated with decreased ergosterol in the fungal membrane [36]. Interestingly, overexpression of the *AFUA_7G01960* gene itself has also been correlated with increased voriconazole resistance [38]. Taken together with bioinformatic analysis, *AFUA_7G01960* is predicted to be a putative transcription factor involved in ergosterol biosynthesis and mutation at L167 likely prevents its activity, thus leading to increased resistance [36]. The last known SNP site we investigated was E180 in *AFUA_2G10600*, a gene encoding the mitochondrial 29.9 KD NADH oxidoreductase subunit of respiratory complex I. The amino acid substitution E180D is present in itraconazole-resistant clinical isolates of *A. fumigatus* [37]. Furthermore, restriction enzyme-mediated insertion of this mutation in the *AFUA_2G10600* gene led to increased itraconazole resistance [71]. This insertion was at a Xhol site, 534 bp from the start codon of the gene. This increase in resistance indicates that intact *AFUA_2G10600* may confer azole susceptibility through mitochondrial NADH metabolism or NAD/NADH redox stress [71]. Complete deletion of the coding region for this 29.9KD subunit was also found to result in itraconazole resistance in the laboratory strain A1163 KU80, increasing from an MIC of 0.25 mg/L to >8 mg/L, which further supports its contribution to itraconazole resistance [37].

Using a Fisher’s Exact test on these 22 mutations, with all 122 strains and using both MIC resistance thresholds (2 mg/L and 4 mg/L), we found only one mutation, L98H in *cyp51A,* to be highly associated with itraconazole and pan-azole resistance (Table 1). Examining all samples with known triazole MIC data, this L98H mutation was found in 21 strains. We also determined using coverage data across the promoter region of *cyp51A* that all 21 strains with the L98H mutation were accompanied with the common 34-bp tandem repeat (Figure S5). A subsequent Fisher’s Exact test was, thus, done after removing strains with the L98H mutation in *cyp51A* (n = 21). Additional Fisher’s exact tests were also conducted after removal of all strains containing the *cyp51A* mutations (n = 64) and conducted again with only Clade II strains (n = 71). However, these additional tests identified no SNPs significantly associated with itraconazole and/or pan-azole resistance. A potential reason why the previously functionally validated sites were not highly associated with triazole resistance in our study is that strain counts for mutation genotypes at these sites were low in our 122-strain sample set and only ranged between 1 and 6 strains, making them unable to meet our criteria (>5% frequency in the population) for inclusion (Table S4).

We further examined the distribution of mutation phenotypes in these functionally validated sites in our three clades, using all 195 strains. Interestingly, for *cyp51A*, the mutation L98H was only present in strains of Clade III (n = 22). For the T289 site, three strains had the mutation T289A and this mutation always accompanied with the substitution Y121F. Furthermore, these three strains were all from Clade III. Mutations in the site G54, specifically G54V (n = 5), G54E (n = 1), G54W (n = 3) and G54R (n = 2), were found mostly in Clade II strains with a rate of 90.91% (10/11). Mutations M220I (n = 2), M220V (n = 1), P216L (n = 4), and F219S (n = 1) were also only found in Clade II strains. For the gene *hmg1*, mutations in this gene were only seen in Clade II strains (n = 7).

Fisher’s Exact tests were also conducted on 37 genes previously found to be overexpressed with triazole exposure using 3230 SNP sites (Table S3). The tests were conducted using both MIC resistance thresholds, 2 mg/L and 4 mg/L. The test conducted with all 122 strains and the MIC threshold set at 2 mg/L found 57 SNPs in or beside 14 genes to be associated with itraconazole resistance. For pan-azole resistance, 11 significantly associated SNPs were found. These SNPs were located in or beside six genes. The test conducted with the MIC threshold of 4 mg/L found the same 57 SNPs in or beside 14 genes to be significantly associated with itraconazole resistance. However, there were slight changes in the SNPs significantly associated with pan-azole resistance when using the 4 mg/L MIC threshold. The 4 mg/L MIC threshold determined 10 SNPs in or beside five genes to be associated with pan-azole resistance. Furthermore, to focus on novel mutations associated with triazole resistance not linked to *cyp51A*, two additional Fisher’s Exact tests were also completed after removing the 21 strains containing the L98H mutation and again after removing the 64 strains with well-known mutations in *cyp51A* (Table S3). Using both MIC thresholds, the results after removal of the 21 strains found three SNPs to be significantly associated with itraconazole resistance. One SNP had already been noted as associated with itraconazole resistance by the first set of Fisher’s Exact test, done prior to the 21-strain removal, which was an intergenic variant in *mfsB*. However, two novel intergenic variants, one in *AFUA_6G01960* and the second in *AFUA_1G16460*, were found to be significantly associated with itraconazole resistance as well. Furthermore, after removal of the 21 strains, no SNPs were found to be significantly associated with pan-azole resistance (Table S3). In terms of the results after removal of the 64 strains, one novel SNP site was found to be significantly associated with triazole differences. Using the MIC threshold of 2 mg/L, the test identified an intergenic variant in *abcA* to be associated with itraconazole resistance (Table S3). A final set of Fisher’s Exact tests were conducted on a clade-level, using only strains in Clade II (n = 71). However, no SNPs sites were significantly associated with triazole resistance using this sample set. The result differences between tests could be due to genetic hitchhiking alongside the resistance polymorphism L98H in *cyp51A* as well as to the reduced sample size, thus decreasing the sample count of certain SNPs and making them unable to meet the Bonferroni-corrected critical *p*-value threshold criteria in these tests.

In about 20% to 70% of triazole-resistant clinical *A. fumigatus* strains, no mutations related to *cyp51A* were observed [29,72]. Molecular assays for the detection of *A. fumigatus* and its *cyp51A* alterations have been produced to provide rapid detection of *cyp51A*-mediated triazole resistance in clinical samples of *A. fumigatus* [72]. However, as shown in our analyses, in many of the triazole-resistant strains, relying on assays targeting only the *cyp51A* mutations would lead to misidentification of these strains as triazole susceptible and cause inappropriate treatment strategies. Indeed, over the last decade, novel triazole resistance mechanisms have been increasingly reported including mutations in *hapE*, *hmg1, yap1,* and *cox10* genes [10,34,35,73]. The wide and growing range of resistance mechanisms seen in *A. fumigatus* demonstrates the high potential this fungus has for stress adaptation, including adaptation to antifungal drug resistance. Delays in the initiation of appropriate antifungal therapy are associated with overall increased mortality. Thus, tools enabling direct detection of resistance using rapid molecular methods can greatly facilitate optimal therapy for individual patients. Together, the putative variants found in this study represent promising candidates for future studies to investigate emerging mechanisms of triazole resistance in *A. fumigatus*. Moreover, these candidate SNPs hold great potential for developing additional diagnostic markers for accurate and rapid identification of triazole resistance in a clinical setting.

## 4. Materials and Methods

### 4.1. Whole Genome Sequences and Strains

Whole-genome sequences for 195 *A. fumigatus* isolates were used in this study. This sample set comprised of 184 whole-genome sequences obtained from the National Center for Biotechnology Information (NCBI) Sequence Read Archive and an additional 12 isolates that were sequenced from our previous study [63]. This strain collection spans 12 countries, across four continents consisting of 61 strains from North America, 1 from South America, 91 from Europe, 40 from Asia, as well as two strains from the International Space Station. In total, 163 of the 195 strains were isolated from a clinical environment, 29 from the natural environment and 3 of unknown sources. Among them, 122 and 123 samples had antifungal susceptibility profiles to itraconazole and voriconazole, respectively. These profiles are recorded as the minimum inhibitory concentrations (MICs) and are presented in Table S1.

### 4.2. Variant Calling

For genome sequence analysis, a modified pipeline from our previous study was used [74]. In brief, FastQC v0.11.5 was used to check for read quality and low-quality sequences were trimmed using Trimmomatic v0.36 [75,76]. The reads were then mapped to the reference *A. fumigatus* strain Af293 (GenBank accession GCA_000002655.1) using the BWA-MEM algorithm v0.7.17 [77]. Duplicate reads were removed using MarkDuplicates in the Picard tool and variants were called using FreeBayes v0.9.21-19 [78,79]. The initial variant filtering was done via vcftools to remove indels, variants with a quality score below 15, and variants with a call rate less than 0.90 [80]. A second filtering step removing multiallelic sites was also conducted using vcftools and this resulting vcf file was named the “soft-filtered” file, which contained 314,999 SNP sites.

### 4.3. Phylogenetic Analysis

To infer evolutionary relationships among the 195 samples, nucleotides of SNP sites were concatenated for each sample and the invariant sites of sequence alignment were removed using RAxML ascertainment bias correction [81]. The maximum likelihood phylogenetic tree was constructed based on 314,999 SNP sites, using the ASC_GTRCAT nucleotide substitution model and 500 bootstrap replicates in RAxML v8.0.25 [81]. The phylogeny was then visualized using iTOL [82]. Strains were assigned into clades based on pairwise SNP comparisons, with a threshold set at 50,000 SNPs.

### 4.4. Genome-Wide Association Study and Linkage Disequilibrium

Variants were annotated with SnpEff v5.0 using the Af293 reference genome annotation to determine functional effects of genetic variants [83]. Highly linked (VIF > 2) SNP markers were removed using PLINK 1.90 beta to ensure uniform sampling of the genome [84]. Association analysis via a mixed linear model was done in TASSEL 5 using two parameters: a population structure defined by 5 principal component vectors, determined based on the scree plot, and a kinship matrix calculated using the Identity by State method (Centered IBS) [85]. To avoid biases due to imbalanced allele frequencies, the minimum allele frequency was also set to 0.05 using TASSEL 5. A total of 21,432 SNP sites remained for conducting the itraconazole GWAS and a total of 21,226 SNP sites for the voriconazole GWAS. A second GWAS was conducted after the removal of strains that contained the L98H mutation in *cyp51A* (n = 21). For the second association analysis, a total of 22,411 and 21,214 SNP sites remained for conducting the itraconazole and voriconazole GWAS, respectively. A third GWAS was also conducted after the removal of strains that contained well-known mutations associated with in *cyp51A* (n = 64). For the third association analysis, a total of 20,176 and 20,278 SNP sites remained for conducting the itraconazole and voriconazole GWAS, respectively. Lastly, we conducted a GWAS examining only the strains from Clade II for itraconazole (n = 71) and voriconazole (n = 72). For the analysis focusing solely on strains from Clade II, a total of 16,702 SNP sites remained for conducting the itraconazole GWAS and a total of 16,782 SNP sites remained for the voriconazole GWAS. Using the results of the GWAS, further association mapping between the top 20 SNPs and all SNPs in the soft-filtered vcf file was conducted using TASSEL 5 to determine additional highly linked SNPs of interest.

## Figures and Tables

**Table 1:** The 44 known mutation sites previously reported to be associated with triazole resistance and results of the Fisher’s Exact tests using 122 *A. fumigatus* strains with known itraconazole and voriconazole MICs.

Gene	Codon	Amino Acid Change	Chromosome—Position (bp)	Fisher’s Exact Test (*p*-Values), MIC ≥ 2 mg/L		Fisher’s Exact Test (*p*-Values), MIC ≥ 4 mg/L		References
				Itraconazole	Pan-Azole	Itraconazole	Pan-Azole	
*cyp51A* *(AFUA_4G06890)*	N22	NA ^1^						[30]
	* F46	Y	CHR 4—1,781,686	4.50 × 10^−3^	3.54 × 10^−2^	4.50 × 10^−3^	2.96 × 10^−2^	[30]
	S52	NA ^1^						[31]
	G54	V, E	CHR 4—1,781,662	1.55 × 10^−1^	1.00	1.55 × 10^−1^	1.00	[30]
		W, R	CHR 4—1,781,663	8.16 × 10^−2^	2.72 × 10^−2^	8.16 × 10^−2^	8.90 × 10^−3^	
	Q88	NA ^1^						[31]
	L98	H	CHR 4—1,781,459	1.19 × 10^−5^	3.33 × 10^−6^	1.19 × 10^−5^	1.47 × 10^−5^	[30]
	V101	NA ^1^						[31]
	Y121	F	CHR 4—1,781,390	2.81 × 10^−1^	2.42 × 10^−1^	2.81 × 10^−1^	9.71 × 10^−2^	[30]
	N125	NA ^1^						[31]
	G138	C	CHR 4—1,781,340	8.09 × 10^−2^	1.00	8.09 × 10^−2^	1.00	[31]
	Q141	NA ^1^						[31]
	H147	Y	CHR 4—1,781,313	1.00	1.00	1.00	1.00	[31]
	F165	NA ^1^						[30]
	* M172	NA ^1^						[30]
	P216	L	CHR 4—1,781,105	5.23 × 10^−1^	4.95 × 10^−1^	5.23 × 10^−1^	2.19 × 10^−1^	[30]
	F219	S	CHR 4—1,781,096	2.91 × 10^−1^	2.44 × 10^−1^	2.91 × 10^−1^	1.04 × 10^−1^	[30]
	M220	I	CHR 4—1,781,092	1.00	1.00	1.00	1.00	[30]
		V	CHR 4—1,781,094	2.87 × 10^−1^	1.00	2.87 × 10^−1^	1.00	
	M236	NA ^1^						[31]
	* N248	NA ^1^						[30]
	* D255	E	CHR 4—1,780,987	6.30 × 10^−1^	1.00	6.30 × 10^−1^	1.00	[30]
	D262	NA ^1^						[30]
	A284	NA ^1^						[30]
	T289	A	CHR 4—1,780,887	2.91 × 10^−1^	2.44 × 10^−1^	2.91 × 10^−1^	1.04 × 10^−1^	[30]
	S297	T	CHR 4—1,780,863	5.26 × 10^−1^	1.00	5.26 × 10^−1^	1.00	[31]
	P394	NA ^1^						[31]
	* E427	K	CHR 4 - 1,780,473	5.00 × 10^−3^	3.69 × 10^−2^	5.00 × 10^−3^	3.11 × 10^−2^	[30]
	Y431	NA ^1^						[30]
	G432	NA ^1^						[30]
	G434	NA ^1^						[30]
	T440	NA ^1^						[30]
	G448	S	CHR 4—1,780,410	1.00	1.00	1.00	4.71 × 10^−1^	[30]
	N479	NA ^1^						[30]
	Y491	NA ^1^						[30]
	F495	I	CHR 4—1,780,269	5.22 × 10^−1^	1.00	5.22 × 10^−1^	1.00	[31]
*cyp51B* *(AFUA_7G03740)*	G457	NA ^1^						[32]
*hapE* *(AFUA_6G05300)*	P88	NA ^1^						[30]
*hmg1* *(AFUA_2G03700)*	F262	NA ^1^						[33]
	S305	P	CHR 2—985,959	5.22 × 10^−1^	4.95 × 10^−1^	5.22 × 10^−1^	2.14 × 10^−1^	[33]
	P309	L	CHR 2—985,972	1.00	1.00	1.00	1.00	[33]
	I412	T, S	CHR 2—986,281	1.56 × 10^−1^	1.17 × 10^−1^	1.56 × 10^−1^	4.34 × 10^−2^	[33]
*erg6* *(AFUA_4G03630)*	A350	NA ^1^						[34]
*cox10* *(AFUA_4G08340)*	R243	NA ^1^						[35]
*AFUA_7G01960*	L167	Stop Gained	CHR 7—531,582	1.00	1.00	1.00	4.66 × 10^−1^	[36]
*AFUA_2G10600*	E180	D	CHR 2—2,714,188	6.39 × 10^−2^	2.33 × 10^−2^	6.39 × 10^−2^	8.79 × 10^−3^	[37]

* The reference strain Af293 contains the *cyp51A* mutations F46Y, M172V, N248T, D255E, and E427K. ^1^ The mutation sites were not found in the soft filtered genotype file, prior to multiallelic site removal.

**Table 2:** Overexpressed genes associated with triazole exposure in *A. fumigatus* from previous RT-qPCR and RNA-seq studies.

Overexpressed Gene Name	Encoded Protein	Fold Change When Exposed to Itraconazole	Fold Change When Exposed to Voriconazole	References
*abcA-1* *(AFUA_1G17440)*	ABC multidrug transporter	7.1	NA	[25]
*abcA-2* *(AFUA_2G15130)*		~6.50	NA	[25]
*abcB* *(AFUA_1G10390)*		~4.50	~5.00–13.00	[25,38]
*abcC* *(AFUA_1G14330)*		~5.50	~5.00–>20.00	[25,38]
*abcD* *(AFUA_6G03470)*		~4.50	~2.00–>20.00	[25,38]
*abcE* *(AFUA_7G00480)*		~1.00	~2.00–>20.00	[25,38]
*atrF* *(AFUA_6G04360)*		31.7	NA	[25]
*mdr1* *(AFUA_5G06070)*		~5.00	~2.00–5.00	[25,38]
*mdr4* *(AFUA_1G12690)*		~4.70	NA	[25]
*AFUA_5G02260*	ABC multidrug transporter, putative	~4.90	NA	[25]
*AFUA_2G11580*	MFS multidrug transporter, putative	14.2	NA	[25]
*mfs56* *(AFUA_1G05010)*		~4.50–700.00	NA	[25]
*mfsA* *(AFUA_8G05710)*	MFS multidrug transporter	~4.70	~1.50–11.00	[25,38]
*mfsB* *(AFUA_1G15490)*		NA	~4.00–18.00	[38]
*mfsC* *(AFUA_1G03200)*		~7.90	~2.50–30.00	[25,38]
*cyp51A* *(AFUA_4G06890)*	14-alpha sterol demethylase	21.00–550.90	NA	[25]
*fbpA* *(AFUA_1G14050)*	F-box domain protein	NA	~ >50.00–600.00	[38]
*aaaA* *(AFUA_7G06680)*	AAA-family ATPase, putative	NA	~2.00–90.00	[38]
*finA* *(AFUA_8G05800)*	C6 zinc finger domain protein	NA	~4.00–40.00	[38]
*AFUA_1G02870*	Transcription factor involved in oxidative stress response, putative	2.48–2.61	NA	[39]
*AFUA_1G04140*	C6 finger domain protein, putative	2.04–2.94	NA	[39]
*AFUA_6G01960*		2.01–3.02	NA	[39]
*AFUA_6G03430*		2.78–2.93	NA	[39]
*fumR* *(AFUA_8G00420)*	C6 zinc finger transcription factor	4.00–4.70	NA	[39]
*AFUA_5G07510*		2.39–3.50	NA	[39]
*AFUA_3G09130*	C6 transcription factor, putative	1.73–2.22	NA	[39]
*AFUA_8G07360*		1.90–1.92	NA	[39]
*cpcA* *(AFUA_4G12470)*	BZIP transcription factor	NA	>1.50–~5.50	[38]
*AFUA_1G16460*	BZIP transcription factor (LziP), putative	1.75–2.12	NA	[39]
*AFUA_7G03910*	C2H2 zinc finger protein	2.50–2.86	NA	[39]
*ace1* *(AFUA_3G08010)*	C2H2 zinc-finger transcription factor, putative	1.66–2.32	NA	[39]
*AFUA_4G13600*		2.30–2.71	NA	[39]
*zfpA* *(AFUA_8G05010)*		NA	~1.50–60.00	[38]
*AFUA_2G01190*	Cu-dependent DNA-binding protein, putative	1.30–2.10	NA	[39]
*AFUA_4G06170*	Predicted DNA-binding transcription factor	3.79–3.89	NA	[39]
*AFUA_5G02655*		2.75–3.84	NA	[39]
*ada* *(AFUA_5G06350)*	DNA repair and transcription factor, putative	1.23–2.05	NA	[39]

**Table 3:** Top 20 significant SNPs obtained from the GWAS that were associated with itraconazole resistance, arranged based on their −log_10_(*p*-values) from the highest to lowest.

Chromosome	Position (bp)	Change	−log_10_(*p*-value)	Gene ID	Annotation	Predicted Effect
1	2,538,614	A to C	6.15	*AFUA_1G09780*	Stomatin family protein	Missense Variant (Asp418Ala)
2	1,845,323	C to T	5.14	*AFUA_2G06330—AFUA_2G07340*	Ubiquitin C-terminal hydrolase, putative—COP9 subunit 3, putative	Intergenic Region
2	1,899,353	C to T	4.58	*AFUA_2G07430—AFUA_2G07440*	DDHD domain protein—Thioesterase family protein	Intergenic Region
3	2,408,041	T to C	4.29	*AFUA_3G09400—AFUA_3G09450*	MFS transporter (Hol1), putative—Alpha/beta fold family hydrolase, putative	Intergenic Region
8	623,331	G to T	4.09	*AFUA_8G02330*	Endoglucanase, putative	Non-coding Transcript Variant
4	3,737,973	C to T	3.66	*AFUA_4G14300—AFUA_4G14310*	Dynamin family GTPase, putative—APH domain-containing protein	Intergenic Region
5	2,063,521	C to A	3.42	*AFUA_5G08150*	ABC bile acid transporter, putative	Missense Variant (His105Gln)
5	2,069,483	G to A	3.23	*AFUA_5G08160—AFUA_5G08170*	Cyclin, putative— Autophagy-related protein 3 (Atg3)	Intergenic Region
3	1,953,910	G to A	3.14	*AFUA_3G07730—AFUA_3G07740*	Uncharacterized protein—Uncharacterized protein	Intergenic Region
6	3,054,001	C to G	3.08	*AFUA_6G12145—AFUA_6G12150*	Uncharacterized protein—BZIP transcription factor (Atf7), putative	Intergenic Region
3	1,266,358	A to G	3.08	*AFUA_3G04310—AFUA_3G05320*	SnoRNA binding protein, putative—C2H2 finger domain protein, putative	Intergenic Region
5	2,069,698	A to G	3.03	*AFUA_5G08160—AFUA_5G08170*	Cyclin, putative— Autophagy-related protein 3 (Atg3)	Intergenic Region
2	1,781,938	G to A	2.99	*AFUA_2G06205—AFUA_2G06220*	Yippee family protein—Zinc knuckle domain protein	Intergenic Region
6	1,353,971	T to C	2.98	*AFUA_6G06350—AFUA_6G06360*	Proteasome subunit alpha type 3, putative—Mating alpha-pheromone (PpgA)	Intergenic Region
4	1,363,615	T to C	2.96	*AFUA_4G04820—AFUA_4G05830*	C-4 methyl sterol oxidase (Erg25), putative—Methylthioribose-1-phosphate isomerase (Mri1)	Intergenic Region
8	635,137	A to G	2.94	*AFUA_8G02350*	Polyketide synthase, putative	Missense Variant (Thr1206Ala)
3	2,316,978	A to G	2.91	*AFUA_3G09090*	RING finger domain protein	Missense Variant (Glu298Gly)
6	2,508,121	A to G	2.91	*AFUA_6G10140—AFUA_6G10150*	C6 transcription factor, putative—Uncharacterized protein	Intergenic Region
2	2,074,852	A to C	2.89	*AFUA_2G08060*	Involucrin repeat protein	Missense Variant (Lys779Thr)
2	2,080,579	T to C	2.89	*AFUA_2G08060*	Involucrin repeat protein	Synonymous Variant (His2640His)

**Table 4:** Top 20 significant SNPs obtained from the GWAS that were associated with voriconazole resistance.

Chromosome	Position (bp)	Change	−log_10_(*p*-value)	Gene ID	Annotation	Predicted Effect
2	1,870,902	G to A	4.69	*AFUA_2G06330—AFUA_2G07340*	Ubiquitin carboxyl-terminal hydrolase—COP9 subunit 3, putative	Intergenic Region
1	975,914	G to A	4.02	*AFUA_1G03370*	Uncharacterized protein	Missense Variant (Ser174Asn)
8	613,458	G to A	3.99	*AFUA_8G02290—AFUA_8G02300*	Uncharacterized protein—FMN-dependent dehydrogenase family protein	Intergenic Region
3	4,040,199	T to C	3.64	*AFUA_3G15350—AFUA_3G15380*	Short chain dehydrogenase family protein, putative—MFS multidrug transporter, putative	Intergenic Region
2	4,689,008	C to T	3.33	*AFUA_2G17600*	Conidial pigment polyketide synthase (Alb1)	Synonymous Variant (Val357Val)
5	564,519	A to C	3.29	*AFUA_5G02210*	Uncharacterized protein	Missense Variant (Met287Arg)
1	2,538,614	A to C	3.29	*AFUA_1G09780*	Stomatin family protein	Missense Variant (Asp418Ala)
2	1,851,010	G to A	2.91	*AFUA_2G06330—AFUA_2G07340*	Ubiquitin carboxyl-terminal hydrolase—COP9 subunit 3, putative	Intergenic Region
8	611,467	C to A	2.85	*AFUA_8G02280*	C6 transcription factor, putative	Missense Variant (Glu79Asp)
2	2,087,757	C to A	2.79	*AFUA_2G08060*	Involucrin repeat protein	Non-coding Transcript Variant
6	3,648,516	T to C	2.78	*AFUA_6G14330*	5-oxo-L-prolinase, putative	Synonymous Variant (Glu131Glu)
1	1,337,273	A to G	2.77	*AFUA_1G04700—AFUA_1G04710*	Ras guanyl-nucleotide exchange factor (RasGEF), putative—Cytoplasmic tRNA 2-thiolation protein 1	Intergenic Region
2	4,805,099	C to T	2.77	*AFUA_2G18070—AFUA_2G18100*	Neutral protease 2—Telomere-associated RecQ helicase, putative	Intergenic Region
2	426,803	C to T	2.75	*AFUA_2G01740*	Sulfate transporter, putative	Synonymous Variant (Ala141Ala)
3	269,388	G to T	2.74	*AFUA_3G01150—AFUA_3G01160*	GPI anchored cell wall protein, putative—Choline monooxygenase, chloroplastic	Intergenic Region
6	2,383,015	C to T	2.70	*AFUA_6G09745—AFUA_6G09760*	Uncharacterized protein—Cytochrome P450 monooxygenase, putative	Intergenic Region
2	420,712	T to C	2.68	*AFUA_2G01710*	GPI anchored protein, putative	Synonymous Variant (Ile294Ile)
7	1,182,007	A to C	2.68	*AFUA_7G05020—AFUA_7G05030*	Uncharacterized protein—Pectin lyase B	Intergenic Region
5	184,363	G to A	2.68	*AFUA_5G00650—AFUA_5G00660*	Uncharacterized protein—Uncharacterized protein	Intergenic Region
2	441,695	C to T	2.67	*AFUA_2G01780*	Small nucleolar ribonucleoprotein complex subunit (Utp15), putative	Synonymous Variant (Val184Val)

**Table 5:** Top 20 significant SNPs obtained from the second GWAS associated with itraconazole resistance, arranged based on their −log_10_(*p*-values) from the highest to lowest.

Chromosome	Position (bp)	Change	−log_10_(*p*-Value)	Gene ID	Annotation	Predicted Effect
1	2,538,614	A to C	5.37	*AFUA_1G09780*	Stomatin family protein	Missense Variant (Asp418Ala)
2	1,845,323	C to T	4.14	*AFUA_2G06330-AFUA_2G07340*	Ubiquitin C-terminal hydrolase, putative—COP9 subunit 3, putative	Intergenic Region
8	623,331	G to T	3.96	*AFUA_8G02330*	Endoglucanase, putative	Non-coding Transcript Variant
2	1,899,353	C to T	3.91	*AFUA_2G07430-AFUA_2G07440*	DDHD domain protein—Thioesterase family protein	Intergenic Region
4	3,737,973	C to T	3.69	*AFUA_4G14300-AFUA_4G14310*	Dynamin family GTPase, putative—APH domain-containing protein	Intergenic Region
5	2,063,521	C to A	3.52	*AFUA_5G08150*	ABC bile acid transporter, putative	Missense Variant (His105Gln)
* 3	267,884	T to G	3.36	*AFUA_3G01140-AFUA_3G01150*	Uncharacterized protein—GPI anchored cell wall protein, putative	Intergenic Region
5	2,069,483	G to A	3.27	*AFUA_5G08160-AFUA_5G08170*	Cyclin, putative— Autophagy-related protein 3 (Atg3)	Intergenic Region
5	2,069,698	A to G	3.13	*AFUA_5G08160-AFUA_5G08170*	Cyclin, putative— Autophagy-related protein 3 (Atg3)	Intergenic Region
* 3	2,389,222	G to A	3.12	*AFUA_3G09400-AFUA_3G09450*	MFS transporter (Hol1), putative—Alpha/beta fold family hydrolase, putative	Intergenic Region
6	3,054,001	C to G	2.99	*AFUA_6G12145-AFUA_6G12150*	Uncharacterized protein—BZIP transcription factor (Atf7), putative	Intergenic Region
* 3	2,414,011	A to G	2.87	*AFUA_3G09480*	15-hydroxyprostaglandin dehydrogenase (NAD(+))	Synonymous Variant (Ser60Ser)
3	1,953,910	G to A	2.84	*AFUA_3G07730-AFUA_3G07740*	Uncharacterized protein—Uncharacterized protein	Intergenic Region
* 6	145,947	T to C	2.84	*AFUA_6G00570-AFUA_6G00580*	Uncharacterized protein—Ankyrin repeat protein	Intergenic Region
* 6	262,795	G to A	2.79	*AFUA_6G01860*	MFS lactose permease, putative	Missense Variant (Val106Met)
* 3	1,883,390	C to A	2.78	*AFUA_3G07510-AFUA_3G07520*	Uncharacterized protein— Exo-beta-1,3-glucanase, putative	Intergenic Region
3	2,316,978	A to G	2.77	*AFUA_3G09090*	RING finger domain protein	Missense Variant (Glu298Gly)
3	1,266,358	A to G	2.76	*AFUA_3G04310-AFUA_3G05320*	SnoRNA binding protein, putative—C2H2 finger domain protein, putative	Intergenic Region
* 1	3,885,980	G to A	2.74	*AFUA_1G14540*	Oxidoreductase, short-chain dehydrogenase/reductase family	Non-coding Transcript Variant
* 6	734,136	G to T	2.72	*AFUA_6G03400-AFUA_6G03430*	Uncharacterized protein—C6 finger transcription factor (FsqA)	Intergenic Region

Unique SNP sites are denoted by asterisks “*” (n = 8).

**Table 6:** Top 20 significant SNPs obtained from the second GWAS associated with voriconazole resistance, arranged based on their −log_10_(*p*-values) from the highest to lowest.

Chromosome	Position (bp)	Change	−log_10_(*p*-Value)	Gene ID	Annotation	Predicted Effect
1	975,914	G to A	3.97	*AFUA_1G03370*	Uncharacterized protein	Missense Variant (Ser174Asn)
2	1,870,902	G to A	3.83	*AFUA_2G06330-AFUA_2G07340*	Ubiquitin carboxyl-terminal hydrolase—COP9 subunit 3, putative	Intergenic Region
8	613,458	G to A	3.71	*AFUA_8G02290-AFUA_8G02300*	Uncharacterized protein—FMN-dependent dehydrogenase family protein	Intergenic Region
* 7	195,144	A to G	3.67	*AFUA_7G00740*	Protein kinase, putative	Missense Variant (Ile188Val)
* 2	3,345,583	A to G	3.53	*AFUA_2G13030*	Phenylalanyl-tRNA synthetase	Missense Variant (Asp343Gly)
* 2	416,242	C to T	3.49	*AFUA_2G01700*	Carbon catabolite derepressing protein kinase (Snf1), putative	Non-coding Transcript Variant
6	3,648,516	T to C	3.41	*AFUA_6G14330*	5-oxo-L-prolinase, putative	Synonymous Variant (Glu131Glu)
6	2,383,015	C to T	3.29	*AFUA_6G09745-AFUA_6G09760*	Uncharacterized protein—Cytochrome P450 monooxygenase, putative	Intergenic Region
* 8	237,297	T to G	3.28	*AFUA_8G01030-AFUA_8G01050*	Uncharacterized protein—Lipase/esterase, putative	Intergenic Region
* 8	379,123	C to T	3.28	*AFUA_8G01480-AFUA_8G01490*	Potassium channel, putative—Endoglucanase, putative	Intergenic Region
* 4	776,628	A to G	3.14	*AFUA_4G02800-AFUA_4G02805*	Haemolysin-III family protein—Asp hemolysin-like protein	Intergenic Region
2	4,689,008	C to T	3.12	*AFUA_2G17600*	Conidial pigment polyketide synthase (Alb1)	Synonymous Variant (Val357Val)
2	2,087,757	C to A	3.12	*AFUA_2G08060*	Involucrin repeat protein	Non-coding Transcript Variant
* 8	292,607	C to T	2.99	*AFUA_8G01250*	GNAT family acetyltransferase, putative	Missense Variant (Arg134Cys)
5	564,519	A to C	2.94	*AFUA_5G02210*	Uncharacterized protein	Missense Variant (Met287Arg)
* 7	1,019,801	A to G	2.94	*AFUA_7G04470-AFUA_7G04480*	Uncharacterized protein—DNA mismatch repair protein (Msh3)	Intergenic Region
* 3	341,035	T to A	2.92	*AFUA_3G01370-AFUA_3G01400*	MFS transporter, putative—ABC multidrug transporter, putative	Intergenic Region
* 3	386,560	T to C	2.90	*AFUA_3G01520*	MFS multidrug transporter, putative	Synonymous Variant (Val170Val)
* 8	1,631,284	G to A	2.87	*AFUA_8G06690-AFUA_8G06700*	Cytochrome P450 alkane hydroxylase—Annexin	Intergenic Region
* 6	2,940,890	T to C	2.86	*AFUA_6G11780-AFUA_6G11790*	Uncharacterized protein—Uncharacterized protein	Intergenic Region

Unique SNP sites are denoted by asterisks “*” (n = 12).

**Table 7:** Top 20 significant SNPs obtained from the third GWAS associated with itraconazole resistance, arranged based on their −log_10_(*p*-values) from the highest to lowest.

Chromosome	Position (bp)	Change	−log_10_(*p*-Value)	Gene ID	Annotation	Predicted Effect
8	635,137	A to G	3.72	*AFUA_8G02350*	Polyketide synthase (PKS), putative	Missense Variant (Thr1206Ala)
8	623,331	G to T	3.50	*AFUA_8G02330*	Endoglucanase, putative	Non-coding Transcript Variant
5	2,069,698	A to G	3.26	*AFUA_5G08160-AFUA_5G08170*	Cyclin, putative—Autophagy-related protein 3 (Atg3)	Intergenic Region
* 5	419,750	A to G	3.11	*AFUA_5G01640-AFUA_5G01650*	Ankyrin repeat protein—bZIP transcription factor (JlbA), putative	Intergenic Region
1	2,538,614	A to C	3.06	*AFUA_1G09780*	Stomatin family protein	Missense Variant (Asp418Ala)
* 8	629,524	G to T	3.01	*AFUA_8G02340-AFUA_8G02350*	Uncharacterized protein—Polyketide synthase, putative	Intergenic Region
* 8	576,158	T to C	2.96	*AFUA_8G02210-AFUA_8G02220*	Alpha-ketoglutarate-dependent taurine dioxygenase—Uncharacterized protein	Intergenic Region
* 5	2,131,740	T to C	2.94	*AFUA_5G08390*	Response regulator, putative (Ssk1)	Synonymous Variant (Lys532Lys)
4	3,737,973	C to T	2.83	*AFUA_4G14300-AFUA_4G14310*	Dynamin family GTPase, putative—APH domain-containing protein	Intergenic Region
* 4	2,539,714	C to A	2.77	*AFUA_4G09770*	Velvet domain-containing protein	Synonymous Variant (Leu193Leu)
1	3,885,980	G to A	2.74	*AFUA_1G14540*	Oxidoreductase, short-chain dehydrogenase/reductase family	Non-coding Transcript Variant
* 3	1,256,445	T to A	2.74	*AFUA_3G04310-AFUA_3G05320*	SnoRNA binding protein, putative— C2H2 finger domain protein, putative	Intergenic Region
* 6	2,141,290	T to C	2.72	*AFUA_6G09000-AFUA_6G09010*	PHD finger domain protein, putative—U1 snRNP splicing complex subunit (Luc7), putative	Intergenic Region
3	2,389,222	G to A	2.70	*AFUA_3G09400-AFUA_3G09450*	MFS transporter (Hol1), putative—Alpha/beta fold family hydrolase, putative	Intergenic Region
* 5	810,835	T to C	2.67	*AFUA_5G03020-AFUA_5G03030*	6 0S ribosomal protein L4, putative— C6 transcription factor, putative	Intergenic Region
* 6	2,891,637	A to G	2.65	*AFUA_6G11620-AFUA_6G11630*	Formyltetrahydrofolate deformylase, putative—FAD-dependent isoamyl alcohol oxidase, putative	Intergenic Region
5	2,063,521	C to A	2.64	*AFUA_5G08150*	ABC bile acid transporter, putative	Missense Variant (His105Gln)
* 8	1,069,676	A to G	2.63	*AFUA_8G04680*	Oxidoreductase, short-chain dehydrogenase/reductase family, putative	Non-coding Transcript Variant
* 1	1,585,001	C to T	2.62	*AFUA_1G00410*	C6 transcription factor, putative	Intragenic Variant
* 4	3,891,318	A to C	2.59	*AFUA_4G14751-AFUA_4G14770*	Uncharacterized protein— Protostadienol synthase (HelA)	Intergenic Region

Unique SNP sites compared to the previous two GWAS analyses are denoted by asterisks “*” (n = 12).

**Table 8:** Top 20 significant SNPs obtained from the third GWAS associated with voriconazole resistance, arranged based on their −log_10_(*p*-values) from the highest to lowest.

Chromosome	Position (bp)	Change	−log_10_(*p*-Value)	Gene ID	Annotation	Predicted Effect
8	613,458	G to A	2.99	*AFUA_8G02290-AFUA_8G02300*	Uncharacterized protein—FMN-dependent dehydrogenase family protein	Intergenic Region
* 5	3,732,385	G to A	2.59	*AFUA_5G14315*	Uncharacterized protein	Synonymous Variant (Phe212Phe)
* 3	246,050	C to A	2.59	*AFUA_3G01060-AFUA_3G01070*	Uncharacterized protein—Tyrosinase, putative	Intergenic Region
* 8	388,274	G to A	2.58	*AFUA_8G01510-AFUA_8G01520*	Uncharacterized protein—Pectinesterase	Intergenic Region
* 5	794,519	G to T	2.57	*AFUA_5G02970*	LCCL domain protein	Synonymous Variant (Thr24Thr)
* 4	2,494,977	G to C	2.55	*AFUA_4G09580*	Major allergen (Aspf2)	Missense Variant (Gly276Ala)
* 8	293,836	G to A	2.52	*AFUA_8G01260*	Uncharacterized protein	Synonymous Variant (Pro383Pro)
* 6	2,379,483	T to C	2.45	*AFUA_6G09745-AFUA_6G09760*	Uncharacterized protein—Cytochrome P450 monooxygenase, putative	Intergenic Region
* 6	2,424,223	C to A	2.44	*AFUA_6G09870*	C6 transcription factor, putative	Missense Variant (Val360Phe)
* 8	791,268	A to G	2.44	*AFUA_8G02870-AFUA_8G03870*	Uncharacterized protein— Uncharacterized protein	Intergenic Region
* 6	2,480,554	C to T	2.43	*AFUA_6G10050-AFUA_6G10060*	Small oligopeptide transporter, OPT family—F-actin-capping protein subunit alpha	Intergenic Region
* 2	1,785,216	G to A	2.42	*AFUA_2G06205-AFUA_2G06220*	Yippee family protein—Zinc knuckle domain protein	Intergenic Region
* 7	1,458,738	C to G	2.37	*AFUA_7G05960*	C2H2 finger domain protein, putative	Missense Variant (Arg759Pro)
* 8	1,548,514	C to T	2.37	*AFUA_8G06410*	MFS multidrug transporter, putative	Synonymous Variant (Arg17Arg)
* 5	2,131,740	T to C	2.37	*AFUA_5G08390*	Response regulator, putative (Ssk1)	Synonymous Variant (Lys532Lys)
* 2	1,774,354	T to C	2.35	*AFUA_2G06205-AFUA_2G06220*	Yippee family protein—Zinc knuckle domain protein	Intergenic Region
* 4	2,539,714	C to A	2.35	*AFUA_4G09770*	Velvet domain-containing protein	Synonymous Variant (Leu193Leu)
* 2	1,787,001	C to T	2.35	*AFUA_2G06205-AFUA_2G06220*	Yippee family protein—Zinc knuckle domain protein	Intergenic Region
2	1,870,902	G to A	2.34	*AFUA_2G06330-AFUA_2G07340*	Ubiquitin carboxyl-terminal hydrolase—COP9 subunit 3, putative	Intergenic Region
* 1	4,762,609	A to G	2.32	*AFUA_1G17410*	Beta-glucosidase, putative	Missense Variant (Val287Ala)

Unique SNP sites compared to the previous two GWAS analyses are denoted by asterisks “*” (n = 18).

**Table 9:** Top 20 significant SNPs obtained from the fourth GWAS associated with itraconazole resistance, arranged based on their −log_10_(*p*-values) from the highest to lowest.

Chromosome	Position (bp)	Change	−log_10_(*p*-Value)	Gene ID	Annotation	Predicted Effect
1	2,538,614	A to C	4.86	*AFUA_1G09780*	Stomatin family protein	Missense Variant (Asp418Ala)
2	1,845,323	C to T	3.21	*AFUA_2G06330—AFUA_2G07340*	Ubiquitin C-terminal hydrolase, putative—COP9 subunit 3, putative	Intergenic Region
8	623,331	G to T	3.04	*AFUA_8G02330*	Endoglucanase, putative	Non-coding Transcript Variant
2	1,899,353	C to T	3.03	*AFUA_2G07430—AFUA_2G07440*	DDHD domain protein—Thioesterase family protein	Intergenic Region
5	2,063,521	C to A	2.91	*AFUA_5G08150*	ABC bile acid transporter, putative	Missense Variant (His105Gln)
3	2,389,222	G to A	2.88	*AFUA_3G09400-AFUA_3G09450*	MFS transporter (Hol1), putative—Alpha/beta fold family hydrolase, putative	Intergenic Region
5	2,069,483	G to A	2.76	*AFUA_5G08160—AFUA_5G08170*	Cyclin, putative— Autophagy-related protein 3 (Atg3)	Intergenic Region
6	3,054,001	C to G	2.76	*AFUA_6G12145—AFUA_6G12150*	Uncharacterized protein—BZIP transcription factor (Atf7), putative	Intergenic Region
5	2,069,698	A to G	2.71	*AFUA_5G08160—AFUA_5G08170*	Cyclin, putative— Autophagy-related protein 3 (Atg3)	Intergenic Region
3	267,884	T to G	2.70	*AFUA_3G01140-AFUA_3G01150*	Uncharacterized protein—GPI anchored cell wall protein, putative	Intergenic Region
* 6	2,895,225	T to C	2.56	*AFUA_6G11620-AFUA_6G11630*	Formyltetrahydrofolate deformylase, putative—FAD-dependent isoamyl alcohol oxidase, putative	Intergenic Region
3	1,953,910	G to A	2.54	*AFUA_3G07730—AFUA_3G07740*	Uncharacterized protein—Uncharacterized protein	Intergenic Region
4	3,737,973	C to T	2.54	*AFUA_4G14300—AFUA_4G14310*	Dynamin family GTPase, putative—APH domain-containing protein	Intergenic Region
* 3	228,628	C to T	2.54	*AFUA_3G00970-AFUA_3G00980*	Uncharacterized protein—MFS transporter Liz1/Seo1, putative	Intergenic Region
* 5	2,042,856	G to A	2.53	*AFUA_5G08050-AFUA_5G08060*	Aminopeptidase P, putative—Importin 13, putative	Intergenic Region
*3	2,456,111	A to G	2.53	*AFUA_3G09630-AFUA_3G09640*	Asparaginyl-tRNA synthetase Slm5, putative—Camp independent regulatory protein	Intergenic Region
* 3	247,848	G to A	2.53	*AFUA_3G01060-AFUA_3G01070*	Uncharacterized protein—Tyrosinase, putative	Intergenic Region
* 3	220,452	G to A	2.52	*AFUA_3G00930*	C6 transcription factor, putative	Synonymous Variant (Ile259Ile)
3	2,408,041	T to C	2.50	*AFUA_3G09400—AFUA_3G09450*	MFS transporter (Hol1), putative—Alpha/beta fold family hydrolase, putative	Intergenic Region
3	2,414,011	A to G	2.48	*AFUA_3G09480*	15-hydroxyprostaglandin dehydrogenase (NAD(+))	Synonymous Variant (Ser60Ser)

Unique SNP sites compared to the previous three GWAS analyses are denoted by asterisks “*” (n = 6).

**Table 10:** Top 20 significant SNPs obtained from the fourth GWAS associated with voriconazole resistance, arranged based on their −log_10_(*p*-values) from the highest to lowest.

Chromosome	Position (bp)	Change	−log_10_(*p*-Value)	Gene ID	Annotation	Predicted Effect
8	613,458	G to A	3.82	*AFUA_8G02290-AFUA_8G02300*	Uncharacterized protein—FMN-dependent dehydrogenase family protein	Intergenic Region
8	611,467	C to A	3.22	*AFUA_8G02280*	C6 transcription factor, putative	Missense Variant (Glu79Asp)
3	246,050	C to A	2.93	*AFUA_3G01060-AFUA_3G01070*	Uncharacterized protein—Tyrosinase, putative	Intergenic Region
* 4	12,352	G to A	2.80	Chr Start*—AFUA_4G00100*	Rhamnogalacturonase, putative	Intergenic Region
* 8	641,537	T to C	2.79	*AFUA_8G02380-AFUA_8G02390*	FAD-dependent monooxygenase, putative—Uncharacterized protein	Intergenic Region
4	2,539,714	C to A	2.75	*AFUA_4G09770*	Velvet domain-containing protein	Synonymous Variant (Leu193Leu)
* 8	331,435	C to A	2.62	*AFUA_8G01340*	MFS sugar transporter, putative	Missense Variant (Leu239Met)
* 5	295,677	C to T	2.62	*AFUA_5G01180*	RAN small monomeric GTPase (Ran), putative	Synonymous Variant(Ser74Ser)
* 5	256,650	T to C	2.61	*AFUA_5G01000*	Oxidoreductase, 2OG-Fe(II) oxygenase family, putative	Missense Variant (Ser110Pro)
* 2	417,623	C to T	2.60	*AFUA_2G01700*	Carbon catabolite derepressing protein kinase (Snf1), putative	Missense Variant (Arg188Gln)
2	426,803	C to T	2.59	*AFUA_2G01740*	Sulfate transporter, putative	Synonymous Variant (Ala141Ala)
* 8	503,790	C to G	2.59	*AFUA_8G01940*	C6 finger domain protein, putative	Missense Variant (Pro261Arg)
2	420,712	T to C	2.55	*AFUA_2G01710*	GPI anchored protein, putative	Synonymous Variant (Ile294Ile)
* 1	1,138,713	A to G	2.53	*AFUA_1G00410*	C6 transcription factor, putative	Intragenic Variant
2	441,695	C to T	2.53	*AFUA_2G01780*	Small nucleolar ribonucleoprotein complex subunit (Utp15), putative	Synonymous Variant (Val184Val)
* 5	3,788,892	C to T	2.53	*AFUA_5G14610*	Carboxypeptidase Y, putative	Missense Variant (Val254Met)
* 5	1,815,994	A to G	2.50	*AFUA_5G07300- AFUA_5G07310*	Electron transfer flavoprotein, beta subunit—DUF500 domain protein	Intergenic Region
* 1	3,306,670	A to C	2.50	*AFUA_1G12540*	TMEM1 family protein, putative	Missense Variant (Phe879Cys)
* 4	1,285,247	G to A	2.50	*AFUA_4G04570*	Uncharacterized protein	Non-coding Transcript Variant
8	388,274	G to A	2.50	*AFUA_8G01510-AFUA_8G01520*	Uncharacterized protein—Pectinesterase	Intergenic Region

Unique SNP sites compared to the previous three GWAS analyses are denoted by asterisks “*” (n = 12).

**Table 11:** Additional non-synonymous SNPs found to be highly linked to the 46 SNP sites obtained by GWAS analyses for itraconazole.

Chromosome	Position	Gene ID	Predicted Effect (Amino Acid Substitution)	Description
2	2,079,605	*AFUA_2G08060*	Missense Variant (Ala2316Ser)	Involucrin repeat protein
2	2,083,296	*AFUA_2G08060*	Missense Variant (Asn3546Ser)	Involucrin repeat protein
2	2,086,695	*AFUA_2G08060*	Missense Variant (Val4679Ala)	Involucrin repeat protein
3	587,378	*AFUA_3G02360*	Missense Variant (Leu413Gln)	Carboxylic ester hydrolase
3	1,604,491	*AFUA_3G06490*	Missense Variant (Gln531Arg)	Uncharacterized protein
3	1,629,278	*AFUA_3G06570*	Missense Variant (Gln77Pro)	Uncharacterized protein
3	1,693,467	*AFUA_3G06800*	Missense Variant (Arg615Thr)	Uncharacterized protein
3	1,700,605	*AFUA_3G06820*	Missense Variant (Lys540Arg)	Oxidoreductase, FAD-binding
3	2,132,951	*AFUA_3G08280*	Missense Variant (Glu28Lys)	Cell cycle regulatory protein (Srw1), putative
3	2,155,356	*AFUA_3G08400*	Missense Variant (Glu393Lys)	SNF2 family helicase/ATPase, putative
3	2,304,691	*AFUA_3G09040*	Missense Variant (Ser13Leu)	Uncharacterized protein
3	2,311,362	*AFUA_3G09070*	Missense Variant (Ile406Thr)	Carboxylesterase, putative
3	2,409,306	*AFUA_3G09450*	Missense Variant (Pro220Leu)	Alpha/beta fold family hydrolase, putative
4	3,875,753	*AFUA_4G14712*	Missense Variant (Pro208Ser)	C6 transcription factor, putative
6	2,583,985	*AFUA_6G10420*	Missense Variant (Gln309Glu)	Uncharacterized protein

**Table 12:** Additional non-synonymous SNPs found to be highly linked to the 62 SNP sites obtained by GWAS analyses for voriconazole.

Chromosome	Position	Gene ID	Predicted Effect (Amino Acid Substitution)	Description
1	976,070	*AFUA_1G03370*	Missense Variant (Ser226Leu)	Uncharacterized protein
1	4,754,138	*AFUA_1G17380*	Missense Variant (Leu226Pro)	3-oxoacyl-(Acyl-carrier-protein) reductase, putative
2	437,241	*AFUA_2G01760*	Missense Variant (Thr1812Ala)	NACHT domain protein
2	541,777	*AFUA_2G02170*	Missense Variant (Ser67Pro)	Nuclear condensin complex subunit (Smc4), putative
5	205,924	*AFUA_5G00730*	Missense Variant (Val814Phe)	H /K ATPase alpha subunit, putative
5	3,290,025	*AFUA_5G12670*	Missense Variant (Phe390Ser)	Nucleoporin (Nup192), putative
6	3,252,789	*AFUA_6G12890*	Missense Variant (Arg878Gly)	Vacuole-associated enzyme activator complex component (Vac14), putative
6	3,330,314	*AFUA_6G13180*	Missense Variant (Ala529Thr)	CECR1 family adenosine deaminase, putative
7	1,457,904	*AFUA_7G05960*	Missense Variant (Arg1037Gln)	C2H2 finger domain protein, putative
7	1,541,519	*AFUA_7G06290*	Missense Variant (Gln666Leu)	NACHT domain protein, putative
8	332,292	*AFUA_8G01340*	Missense Variant (Met524Ile)	MFS sugar transporter, putative

**Table 13:** Highly linked significant SNP sites associated with triazole resistance determined using Fisher’s Exact tests (n = 122).

Chromosome	Position (bp)	Gene ID	Predicted Effect (Amino Acid Substitution)	Fisher’s Exact Test (*p*-Values), MIC ≥ 2 mg/L		Fisher’s Exact Test (*p*-Values), MIC ≥ 4 mg/L	
				Itraconazole	Pan-Azole	Itraconazole	Pan-Azole
1	976,070	*AFUA_1G03370*	Missense Variant (Ser226Leu)	2.37 × 10^−5^ *	8.10 × 10^−6^ *	2.37 × 10^−5^ *	5.48 × 10^−6^ *
1	4,754,138	*AFUA_1G17380*	Missense Variant (Leu226Pro)	8.40 × 10^−6^ *	5.57 × 10^−5^ *	8.40 × 10^−6^ *	9.21 × 10^−5^ *
3	2,304,691	*AFUA_3G09040*	Missense Variant (Ser13Leu)	3.92 × 10^−4^ *	3.82 × 10^−3^	3.92 × 10^−4^ *	2.41 × 10^−3^
3	2,311,362	*AFUA_3G09070*	Missense Variant (Ile406Thr)	3.74 × 10^−4^ *	1.99 × 10^−3^	3.74 × 10^−4^ *	2.41 × 10^−3^
3	2,409,306	*AFUA_3G09450*	Missense Variant (Pro220Leu)	2.79 × 10^−4^ *	7.05 × 10^−5^ *	2.79 × 10^−4^ *	1.64 × 10^−4^ *

* Statistically significant association between SNP and antifungal resistance.

**Table 14:** Highly linked significant SNP sites associated with triazole resistance determined using Fisher’s Exact tests after removing the 21 strains with the L98H mutation in *cyp51A* (n = 101).

Chromosome	Position (bp)	Gene ID	Predicted Effect (Amino Acid Substitution)	Fisher’s Exact Test (*p*-Values), MIC ≥ 2 mg/L		Fisher’s Exact Test (*p*-Values), MIC ≥ 4 mg/L	
				Itraconazole	Pan-Azole	Itraconazole	Pan-Azole
1	976,070	*AFUA_1G03370*	Missense Variant (Ser226Leu)	3.20 × 10^−3^	4.79 × 10^−4^ *	3.20 × 10^−3^	1.84 × 10^−4^ *
1	4,754,138	*AFUA_1G17380*	Missense Variant (Leu226Pro)	3.15 × 10^−4^ *	2.12 × 10^−3^	3.15 × 10^−4^ *	1.41 × 10^−3^
3	2,304,691	*AFUA_3G09040*	Missense Variant (Ser13Leu)	1.06 × 10^−5^ *	7.39 × 10^−5^ *	1.06 × 10^−5^ *	6.19 × 10^−5^ *
3	2,311,362	*AFUA_3G09070*	Missense Variant (Ile406Thr)	5.07 × 10^−6^ *	3.47 × 10^−5^ *	5.07 × 10^−6^ *	6.19 × 10^−5^ *
7	1,541,519	*AFUA_7G06290*	Missense Variant (Gln666Leu)	1.08 × 10^−3^	4.35 × 10^−4^ *	1.08 × 10^−3^	1.29 × 10^−3^

* Statistically significant association between SNP and antifungal resistance.

**Table 15:** Highly linked significant SNP sites associated with triazole resistance determined using Fisher’s Exact tests after removing the 64 strains with the mutations in *cyp51A* (n = 58).

Chromosome	Position (bp)	Gene ID	Predicted Effect (Amino Acid Substitution)	Fisher’s Exact Test (*p*-Values), MIC ≥ 2 mg/L		Fisher’s Exact Test (*p*-Values), MIC ≥ 4 mg/L	
				Itraconazole	Pan-Azole	Itraconazole	Pan-Azole
1	4,754,138	*AFUA_1G17380*	Missense Variant (Leu226Pro)	1.87 × 10^−5^ *	1.25 × 10^−4^ *	1.87 × 10^−5^ *	2.91 × 10^−4^ *
3	2,304,691	*AFUA_3G09040*	Missense Variant (Ser13Leu)	8.33 × 10^−5^ *	2.33 × 10^−5^ *	8.33 × 10^−5^ *	3.10 × 10^−5^ *
3	2,311,362	*AFUA_3G09070*	Missense Variant (Ile406Thr)	8.33 × 10^−5^ *	2.33 × 10^−5^ *	8.33 × 10^−5^ *	3.10 × 10^−5^ *
7	1,541,519	*AFUA_7G06290*	Missense Variant (Gln666Leu)	1.18 × 10^−3^	3.64 × 10^−4^ *	1.18 × 10^−3^	5.03 × 10^−4^ *

* Statistically significant association between SNP and antifungal resistance.

**Table 16:** Highly linked significant SNP sites associated with triazole resistance determined using Fisher’s Exact tests and strains in Clade II (n = 71).

Chromosome	Position (bp)	Gene ID	Predicted Effect (Amino Acid Substitution)	Fisher’s Exact Test (*p*-Values), MIC ≥ 2 mg/L		Fisher’s Exact Test (*p*-Values), MIC ≥ 4 mg/L	
				Itraconazole	Pan-Azole	Itraconazole	Pan-Azole
1	4,754,138	*AFUA_1G17380*	Missense Variant (Leu226Pro)	1.68 × 10^−6^ *	4.81 × 10^−5^ *	1.68 × 10^−6^ *	4.83 × 10^−5^ *
3	2,304,691	*AFUA_3G09040*	Missense Variant (Ser13Leu)	2.59 × 10^−5^ *	8.51 × 10^−5^ *	2.59 × 10^−5^ *	1.73 × 10^−5^ *
3	2,311,362	*AFUA_3G09070*	Missense Variant (Ile406Thr)	1.17 × 10^−5^ *	3.48 × 10^−5^ *	1.17 × 10^−5^ *	1.73 × 10^−5^ *
7	1,541,519	*AFUA_7G06290*	Missense Variant (Gln666Leu)	3.21 × 10^−3^	5.88 × 10^−4^*	3.21 × 10^−3^	2.85 × 10^−4^*

* Statistically significant association between SNP and antifungal resistance.

## Data Availability

Accession numbers for all 195 isolates used in this study are available and listed in Supplementary Table S1.

## Supplementary Materials

The following are available online at https://www.mdpi.com/article/10.3390/pathogens10060701/s1, Figure S1: Quantile–quantile (Q-Q) plots from the GWAS for (A) itraconazole and (B) voriconazole, Figure S2: Quantile–quantile (Q-Q) plots from the second GWAS analysis, after removal of the 21 strains containing the L98H mutation in *cyp51A*, for (A) itraconazole and (B) voriconazole, Figure S3: Quantile–quantile (Q-Q) plots from the third GWAS analysis, after removal of the 64 strains containing the well-known mutations in *cyp51A*, for (A) itraconazole and (B) voriconazole, Figure S4: Quantile–quantile (Q-Q) plots from the fourth GWAS analysis, focusing on strains from Clade II, for (A) itraconazole and (B) voriconazole, Figure S5: Coverage at promoter region of *cyp51A* from position 1,782,000 to 1,782,200 bp for the 21 strains with L98H mutation and known triazole MIC values, Table S1: Additional information on the total 195 *Aspergillus fumigatus* genomes, Table S2: Overexpressed genes with itraconazole and voriconazole exposure determined through previous RT-qPCR and RNA-seq analyses, Table S3: Significant single-nucleotide polymorphisms located in or near genes overexpressed with triazole exposure.
